# Changes in N-Transforming Archaea and Bacteria in Soil during the Establishment of Bioenergy Crops

**DOI:** 10.1371/journal.pone.0024750

**Published:** 2011-09-14

**Authors:** Yuejian Mao, Anthony C. Yannarell, Roderick I. Mackie

**Affiliations:** 1 Energy Biosciences Institute, University of Illinois, Urbana, Illinois, United States of America; 2 Institute for Genomic Biology, University of Illinois, Urbana, Illinois, United States of America; 3 Department of Natural Resources and Environmental Sciences, University of Illinois, Urbana, Illinois, United States of America; 4 Department of Animal Sciences, University of Illinois, Urbana, Illinois, United States of America; Argonne National Laboratory, United States of America

## Abstract

Widespread adaptation of biomass production for bioenergy may influence important biogeochemical functions in the landscape, which are mainly carried out by soil microbes. Here we explore the impact of four potential bioenergy feedstock crops (maize, switchgrass, *Miscanthus X giganteus*, and mixed tallgrass prairie) on nitrogen cycling microorganisms in the soil by monitoring the changes in the quantity (real-time PCR) and diversity (barcoded pyrosequencing) of key functional genes (*nifH*, bacterial/archaeal *amoA* and *nosZ*) and 16S rRNA genes over two years after bioenergy crop establishment. The quantities of these N-cycling genes were relatively stable in all four crops, except maize (the only fertilized crop), in which the population size of AOB doubled in less than 3 months. The nitrification rate was significantly correlated with the quantity of ammonia-oxidizing archaea (AOA) not bacteria (AOB), indicating that archaea were the major ammonia oxidizers. Deep sequencing revealed high diversity of *nifH*, archaeal *amoA*, bacterial *amoA*, *nosZ* and 16S rRNA genes, with 229, 309, 330, 331 and 8989 OTUs observed, respectively. Rarefaction analysis revealed the diversity of archaeal *amoA* in maize markedly decreased in the second year. Ordination analysis of T-RFLP and pyrosequencing results showed that the N-transforming microbial community structures in the soil under these crops gradually differentiated. Thus far, our two-year study has shown that specific N-transforming microbial communities develop in the soil in response to planting different bioenergy crops, and each functional group responded in a different way. Our results also suggest that cultivation of maize with N-fertilization increases the abundance of AOB and denitrifiers, reduces the diversity of AOA, and results in significant changes in the structure of denitrification community.

## Introduction

Bioenergy derived from cellulosic ethanol is a potential sustainable alternative to fossil fuel-based energy, since the energy from green plants is renewable and largely carbon neutral in comparison to fossil fuel combustion. Perennial grasses, such as switchgrass (*Panicum virgatum*) and *Miscanthus×giganteus*, with large annual biomass production potential, are proposed as biofuel feedstocks that can maximize ethanol production without adversely affecting the market for food crops (e.g. maize). However, our knowledge of the impacts of various bioenergy feedstock production systems on the soil microbial ecosystem is still very limited. The chemistry of perennial crop residues and plant root exudates may stimulate or inhibit the growth and activity of different fractions of the soil microbial community, and thus the planting of different crops can result in distinct microbial communities [1], [2], [3]. Differences in management techniques between traditional row-crop agriculture and perennial biomass feedstocks represent different soil disturbance regimes, altered water use, differing rates of fertilizer application, etc., and these should have a direct impact on soil microbial dynamics, subsequently influencing the terrestrial biogeochemical cycles. In particular, we predict that the cultivation of high nitrogen-use efficiency perennial grasses will result in altered nitrogen-transforming microbial communities in comparison to those found under N-fertilized maize.

The biological nitrogen cycle is one of the most important nutrient cycles in the terrestrial ecosystem. It includes four major processes: nitrogen fixation, mineralization (decay), nitrification and denitrification. Because many of the microorganisms responsible for these processes are recalcitrant to laboratory cultivation, previous studies of the distribution and diversity of nitrogen-transforming microorganisms have employed cultivation-independent techniques targeting functional genes: *nifH*, *amoA* and *nosZ* genes, which encode the key enzymes in nitrogen fixation, ammonia oxidization and complete denitrification, respectively [4], [5], [6], [7], [8], [9].

Biological nitrogen fixation, which converts atmospheric N_2_ into ammonium that is available to organisms, is an important natural input of available nitrogen in many terrestrial habitats [10]. Although nitrogen fixation in terrestrial ecosystems is thought to be mainly carried out by the symbiotic bacteria in association with plants, free-living diazotrophs in soils can play important roles in N cycling in a number of ecosystems [11], [12]. In average, 2–3 kg N ha^−1^ year^−1^ could be imported by free living N-fixers [13]. Various field experiments have shown that the biomass yield of one candidate biofuel feedstock crop, *Miscanthus×giganteus*, is not significantly increased by the addition of mineral N fertilizer [14]. The lack of response to nitrogen fertilization and the high biomass production suggest that biological nitrogen fixation may play an important role in supplying the nitrogen needs of *Miscanthus* [15]. Plant species have previously been shown to have a significant effect on the composition of diazotrophs in the field; for example, diazotroph diversity is higher in soil under *Acacia tortilis* ssp. *raddiana* (a leguminous tree) than *Balanites aegyptiaca* (a non-leguminous tree) [16]. Plant genotype also has a strong effect on the rhizosphere diazotrophs of rice [17]. Agronomic practices can also influence soil diazotrophs, e.g. application of N-fertilization can reduce the diversity of diazotrophs [17]. Therefore, we hypothesize that the cultivation of maize with inorganic N-fertilizer will reduce the abundance and diversity of diazotrophs in the soil ecosystem, while biofuel feedstocks receiving little or no N-fertilizer (e.g. *Miscanthus*) will encourage the development of active diazotrophic communities.

Nitrification, which converts ammonium to nitrate, includes two steps: ammonia oxidation to nitrite, and nitrite oxidation to nitrate. The production of nitrate in soil not only supplies nutrition for plants, but it can also mobilize nitrogen to groundwater through nitrate leaching. Ammonia oxidation is the first and rate-limiting step of nitrification [18]. It is typically thought to be carried out by a few groups in β- and γ- Proteobacteria, referred to as ammonia-oxidizing bacteria (AOB) [18]. However, recent environmental metagenomic analyses revealed that ammonia monooxygenase α-subunit (*amoA*) genes are also present in archaea (AOA) [19], and archaeal *amoA* has been shown to be widespread in many environments, e.g. soils, hot springs and marine water [6], [19], [20], [21], [22]. Recent work has found that AOA can be up to 3000 times more abundant in soil than AOB [22], [23], [24], meaning that AOA are the most abundant ammonia oxidizing organisms in soil ecosystems [25]. The soil ammonia oxidizing community is known to be influenced by plant types and management, but different segments of this community respond differently [24], [26], [27]. For example, the abundance and composition of AOB is significantly altered by long-term fertilization, but AOA are rarely affected [24], [27]. The nitrification activity in soil ecosystems is known to be correlated with the abundances and structures of ammonia oxidizers [24], [28], [29]. We therefore hypothesize that different biofuel cropping systems, especially those that rely on N-fertilization, will influence the composition of ammonium oxidizers in soil, with potential consequences for nitrification rates.

Denitrification, which reduces nitrate to N_2_ gas, is carried out by a diverse group of microorganisms belonging to more than 60 genera of bacteria, archaea, and some eukaryotes [30]. Complete denitrification involves four steps: NO_3_^−^→NO_2_^−^→NO→N_2_O→N_2_. The enzyme nitrous oxide reductase (encoded by *nosZ*) that reduces N_2_O to N_2_ is essential for complete conversion of NO_3_^−^ to N_2_. Approximately 17 Tg N is estimated to be lost from the land surface through denitrification every year [31]. It is known that the structure and activity of denitrifiers in the terrestrial ecosystem could be significantly influenced by the plant species [7], [29], [32]. In a study of a maize-cropped field, it was found that organic or mineral fertilizer applications could affect both the structure and activity of the denitrifying community in the long term, with changes persisting for at least 14 months [33]. The potential denitrification rate was found to be significantly correlated to the denitrifier density, as estimated by the quantification of *nosZ* gene copy numbers [34]. Denitrification releases mineralized nitrogen in the soil ecosystem to the atmosphere, and thus, the balance between denitrification and N-fixation, can determine the biologically available N for the biosphere (Arp, 2000).

It is known that plant species can change the soil microbial community [1]. However, while much previous work has examined the microbial community differences between the established crops [7], [29], [34], [35], [36], less is known about how microbial communities in the agricultural soils develop during the transition from one cropping system to another (e.g. annual row crops to perennial biofuel feedstocks). Thus, to improve our knowledge of the effects of bioenergy feedstock production on the complex N-cycling microbial communities of terrestrial ecosystems, we followed the changes in soil microbial communities during a two-year establishment period of maize, switchgrass, *Miscanthus×giganteus*, and mixed tallgrass prairie. We monitored the abundance of key genes for nitrogen fixation, ammonia oxidation and complete denitrification (*nifH*, bacterial/archaeal *amoA* and *nosZ*) as well as the structural changes of these N-cycling genes and bacterial/archaeal 16S rRNA genes using real-time PCR and barcoded pyrosequencing methods respectively.

## Materials and Methods

### Study site and sampling

The experiment was conducted at the Energy Biosciences Institute's Energy Farm located southwest of Urbana, Illinois, USA. Miscanthus (*Miscanthus*×*giganteus*, MG), switchgrass (*Panicum virgatum*, PV), maize (*Zea mays*, ZM) and restored tallgrass prairie (used as control, NP) were planted in the spring of 2008. Miscanthus was replanted in the spring of 2009 due to its poor growth in 2008. Each crop was planted in a randomized block design, with a 0.7-ha plot of each crop randomly positioned within four blocks (n = 4 for each crop). Samples were collected in April 2008, before planting of these crops, in order to characterize the background soil microbial communities. Bulk soil samples were collected at monthly intervals during the growing seasons (June–September 2008 and 2009). 10 soil cores (0–10 cm depth, 1.8 cm diameter) were collected from each plot and homogenized in an ethanol-sanitized, plastic bucket. About 60 g of the well-mixed soil was then subsampled into a 50 mL tube for each plot, and kept on ice until brought to the lab and stored in a −80°C freezer. In total, 112 soil samples were collected. Following standard agricultural practices, only maize was fertilized (17 g/m^2^ in 2008, 20 g/m^2^ in 2009) with a mixture of urea, ammonia and nitrate (28% UAN). Herbicides were applied in these crops except the restored tallgrass prairie: 1.56 l/ha of Roundup (only applied in 2008) and 4.13 l/ha of Lumax for maize; 1.37 l/ha of 2,4-Dichlorophenoxyacetic acid for Miscanthus; 1.37 l/ha of 2,4-Dichlorophenoxyacetic acid for switchgrass in 2008.

### DNA extraction and purification

Soil samples were freeze-dried overnight until completely dry and then manually homogenized with a sterile screwdriver. DNA was extracted from 0.3 g soil using the FastDNA SPIN Kit For Soil (MP Biomedicals) according to manufacturer's protocol. Extracted DNA was then purified using CTAB. DNA concentrations were determined using the Qubit quantification platform with Quant-iT™ dsDNA BR Assay Kit (Invitrogen). DNA was diluted to 10 ng/µL and stored in −80°C freezer for the following molecular applications.

### Real-time PCR

The abundances of *nifH*, archaeal *amoA*, bacterial *amoA* and *nosZ* genes in all the soil samples were quantified using real-time PCR. Quantitative real-time PCR was performed according to the methods modified from previous studies: *nifH* (as a measure of N-fixing bacteria) used primers PolF (5′- TGC GAY CCS AAR GCB GAC TC-3′) and PolR (5′-ATS GCC ATC ATY TCR CCG GA-3′) [5]; archaeal *amoA* (as a measure of ammonia-oxidizing archaea) used primers Arch-amoAF (5′-STA ATG GTC TGG CTT AGA CG-3′) and Arch-amoAR (5′-GCG GCC ATC CAT CTG TAT GT-3′) [6]; bacterial *amoA* (as a measure of ammonia-oxidizing bacteria) used primers amoA-1F (5′-GGG GTT TCT ACT GGT GGT-3′) and amoA-2R (5′-CCC CTC KGS AAA GCC TTC TTC-3′) [4]; and *nosZ* (as a measure of denitrification bacteria) used primers nosZ-F (5′-CGY TGT TCM TCG ACA GCC AG-3′) [37] and nosZ 1622R (5′-CGS ACC TTS TTG CCS TYG CG-3′) [7]. Purified PCR products from a common DNA mixture (equal amounts of DNA from all samples collected in August of 2008 and 2009) were used to prepare sample-derived quantification standards as previously described [38]. The copy number of gene in each standard was calculated by DNA concentration (ng/µL, measured by Qubit) divided by the average molecular weight (estimated based on the barcoded-pyrosequencing results) of that gene. In comparison to using a clone (plasmid) as standard, this method avoids the difference of PCR amplification efficiency between standards and samples caused by the different sequence composition in the PCR templates (single sequence in a plasmid for the standard vs. mixture of thousands of sequences in a soil sample). The 10 µL reaction mixture contained 5 µL of 2× Power SYBR Green Master Mix (Applied Biosystems), 0.5 µL of BSA (10 mg/mL, New England Biolabs), 0.4 µL of each primer (10 µM) and 5 ng of DNA template. Real-time amplification was performed in an ABI Prism 7700 Sequence Detector with MicroAmp Optical 384-Well Reaction Plate and Optical Adhesive Film (Applied Biosystems) using the following program: 94°C for 5 min; 40 cycles of 94°C for 45 s, 56°C for 1 min (54°C for *nifH* gene), 72°C for 1 min. A dissociation step was added at the end of the qPCR to assess amplification quality. The specificity of the PCR was further evaluated by running twenty randomly selected samples (for each gene) on a 1% (w/v) agarose gel. The corresponding real-time PCR efficiency for each of these genes was estimated based on a two-fold serial dilution of the common DNA mixture described above. The qPCR efficiency (*E*) was calculated according to the equation E = 10^[−1/slope]^ [39]. Triplicate qPCR repetitions were performed for each of the gene for all the samples. The real-time PCR amplification efficiency of *nifH*, archaeal *amoA*, bacterial *amoA* and *nosZ* genes was 1.90±0.01, 1.90±0.06, 1.76±0.01 and 1.82±0.01, respectively. The *R^2^* of all these standard curves was higher than 0.99. The detection limit of this real-time PCR assay was 10 copies/µL.

The copy numbers of these genes per gram of dry soil was calculated by the copy numbers of the gene per ng of DNA multiplied by the amount of DNA contained in each gram of dry soil. The quantities of these genes were corrected assuming a DNA extraction efficiency of 30% [40], [41].

### T-RFLP

The soil samples collected from four replicated (blocks) plots of the four crops prior to planting, and then August of establishment years 1 and 2 (2008 and 2009; 48 samples in total) were analyzed by terminal restriction fragment length polymorphism (T-RFLP). The *nifH*, archaeal *amoA*, bacterial *amoA*, *nosZ* gene were amplified from these samples with FAM-labeled (on forward primer) primers PolF/R, Arch-amoAF/R, amoA-1F/2R and nosZ-F/1622R (see Table S1). The 16S rRNA gene was amplified with 8F (5′-FAM-AGAGTTTGATCMTGGCTCAG-3′) and 1492R (5′-GGTTACCTTGTTACGACTT-3′) [42], [43]. The PCR reaction mixture (25 µl) contained 5 µl GoTaq Flexi Buffer (5×), 2 µl MgCl_2_ (25 mM), 0.25 µl DNA Polymerase (5 U/µl, Promega, Madison, Wis.), 1.25 µl BSA (10 mg/ml), 1 µl forward primer (10 µM), 1 µl reverse primer (10 µM), 1.25 µl dNTP Mix (10 mM), 2 µl DNA template (10 ng/µl). The PCR reaction was performed in a thermo cycler (BioRad, Hercules, CA) using a 5-min heating step at 94°C followed by 30 cycles of denaturing at 94°C for 1 min, annealing at 60°C (54°C for *nifH*) for 45 s, and extension at 72°C for 1 min, with a final extension step of 5 min at 72°C. The PCR products were purified by QIAquick PCR Purification Kit (QIAGEN) and digested at 37°C overnight in a 20-µl mixture containing 2 µl NEB Buffer (10×), 0.5 µl AluI/HhaI (20 U/µl) and 5 µl PCR product. 5 µl of the digested product was sent to the Core Sequencing Facility (University of Illinois at Urbana-Champaign) for fragment analysis. ROX1000 was used as inner standard. T-RFLP profiles were analyzed by GeneMarker (v 1.85) according to the manufacturer's instruction. Fragments with sizes between 50 bp and the length of the PCR products and peak area >500 were selected for T-RFLP profile statistical analysis. The profile data were normalized by calculating the relative abundance (percentage) of each fragment (individual peak area divided by the total peak area).

### Barcoded pyrosequencing

The same samples used in T-RFLP were also used in barcoded pyrosequencing. The four replicated samples collected from the same crop at the same time were combined in to one composite sample for the construction of sequence libraries. Altogether, nine samples (one sample for background soil [mixed from the soils from all the plots before planting these crops], four samples from each of the different crops at Aug 2008, and four from Aug 2009) were obtained. Furthermore, all of these N-transforming genes of the sample collected from MG at the end of the second year (MG2) were sequenced twice in two different lanes to estimate the variation of the sequencing method. The 16S rRNA gene of ZM2 was also sequenced twice. Details of primers and PCR conditions used in the study are listed in Table S1.

The *nifH*, archaeal *amoA*, bacterial *amoA*, *nosZ* and 16S rRNA genes (V4–V5 region) were amplified using the barcode primers PolF/R, Arch-amoAF/R, amoA-1F/2R, nosZ-F/1622R and U519F/U926R, respectively (primers are shown in Table S1). The primers (HPLC purified, Integrated DNA Technologies) were designed as 5′-Fusion Primer+barcode+gene specific primer-3′ (Fusion Primer A, 5′- CGTATCGCCTCCCTCGCGCCATCAG-3′; Fusion Primer B, 5′-CTATGCGCCTTGCCAGCCCGCTCAG-3′). The PCR conditions were optimized and primers with appropriate barcodes (10 bp) were selected. The barcodes used for each primer are described in NCBI SRA, with accession number SRA023700. The 50 µL PCR mixture contained 10 µL of 5× Phusion HF Buffer (Phusion GC Buffer was used for bacterial *amoA* gene amplification, both buffer contains 7.5 mM MgCl_2_), 1 µL of 10 mM dNTPs, 2.5 µL of 10 mg/mL BSA, 0.5 µL of 2 U/µL Phusion Hot Start DNA Polymerase (FINNZYMES), 4 µL of 10 µM forward/reverse primer mixture and 4 µL of 10 ng/µL DNA templates. 1 µL of 100% DMSO was supplemented into the PCR mixture in bacteria *amoA* gene (GC rich) amplification. The PCR amplification was performed in a thermal cycler (BioRad, Hercules, CA) using the program 98°C for 2 min; 30 cycles of 98°C for 10 s, 60°C (54°C for *nifH* gene and 56°C for bacterial *amoA* gene) for 30 s, 72°C for 20 s; 72°C for 5 min. The PCR product was first checked on a 1.2% w/v agarose gel, and then purified by QIAquick PCR Purification Kit (QIAGEN). The DNA concentration of the purified PCR product was measured by Qubit Fluorometer using the Quant-iT™ dsDNA BR Assay Kit (Invitrogen) according to the manual. PCR products of the same gene, to be run together in the same lane (1/16 plate) in 454 sequencing, were mixed in equal mole amounts and run on a 2% w/v agarose gel. The target bands were cut from the gel and purified by QIAquick Gel Extraction Kit (QIAGEN). The DNA concentrations of the purified PCR products were measured by Qubit Fluorometer and adjusted to 50 nM. The *nifH*, archaeal *amoA*, bacterial *amoA* and *nosZ* genes PCR products were then mixed in equal mole amounts and sequenced on a Genome Sequencer FLX Instrument (Roche) using GS FLX Titanium series reagents. The 16S rRNA gene was run in a separate lane.

### Sequence analysis

Sequences were first extracted from the raw data according the Genome Sequencer Data Analysis Software Manual (Software Version 2.0.00, October 2008) by the sequencing center (Roy J. Carver Biotechnology Center, University of Illinois at Urbana-Champaign). The sequences with low quality (length <50 bp, which ambiguous base ‘N’, and average base quality score <20, for detail see manual) were removed. The sequences that fully matched with the barcodes were selected and distributed to separate files for each of the different genes, after removal of the barcode, using RDP Pipeline Initial Process (http://pyro.cme.msu.edu/). For each gene, the sequences that didn't match with the gene specific primers or had a read length shorter than 350 bp were removed. The sequences that matched with the reverse primer were converted to their reverse complement counterparts using BioEdit to make all the sequences forward-oriented.

The 16S rRNA gene sequences were aligned by NAST (Greengenes). The sequences with significant matched minimum length <300 and identity <75% were removed. The aligned 16S rRNA gene sequences were used for chimera check using Bellerophon method in Mothur [44]. Distance matrices were calculated by ARB using the neighbor joining method [45]. A lane mask was used in calculating the 16S rRNA gene sequences to filter out the hyper variable regions. Operational Taxonomic Units (OTUs) were then classified using a 97% nucleotide sequence similarity cutoff and rarefaction curves were constructed based on the distance matrices (both of nucleotide and amino acid sequences) using DOTUR [46]. The phylogenetic affiliation of each 16S rRNA gene sequence was analyzed by RDP CLASSIFIER (http://rdp.cme.msu.edu/) using confidence level of 80%.

The 16S rRNA gene sequences were also processed by QIIME pipeline and denoised by Denoiser V0.91 according to the manual [47], [48]. The results were compared to that obtained by RDP pipeline. In total, 26,431 valid sequences were obtained after denoising using QIIME, which is 12.2% less than that obtained by RDP pipeline (without denoising). Using the 97% similarity cutoff, 8,568 OTUs were obtained, which is 4.7% lower than that observed by RDP pipeline. After random re-sampling to the same sequence depth (1789 sequences per sample) using Daisy_chopper (http://www.genomics.ceh.ac.uk/GeneSwytch/Tools.html), the number of OTUs for each sample obtained by two different processing methods (QIIME, denoised and RDP, non-denoised) was compared (Fig. S1). The estimated number of OTUs after denoising was similar to that obtained by RDP pyrosequencing pipeline (without denoising), showing that the denoising process had a very limited influence on our diversity analysis. The data reported in this paper was analyzed using RPD pipeline described in the previous paragraph.

The *nifH*, archaeal *amoA*, bacterial *amoA* and *nosZ* genes sequences were blasted against a non-redundant protein sequence database (download from NCBI) using BLASTX with an *E*-value cutoff of 0.001. The top 10 closest matches of each sequence were estimated using a custom made Perl script to remove possible chimeras and sequences with sequencing errors causing frameshifts. Sequences with different regions matching the same sequence in the database but with different frame positions were considered to be frameshifts. Sequences that matched two or more different origin sequences were classified as chimeras. The nucleotide sequences of *nifH*, archaeal *amoA*, bacterial *amoA* and *nosZ* genes were translated into amino acid by Geneious (http://www.geneious.com/) based on the frame positions obtained from BLASTX. The redundant sequences (identical sequences) were removed using CD-Hit [49], and the representatives with longest length were selected for following phylogenetic analysis. Both of the nucleotide and amino acid sequences of these N-cycling genes were aligned by MUSCLE 3.7 [50] using program default settings. Operational Taxonomic Units (OTUs) were then classified and rarefaction curves were constructed based on the distance matrices (both of nucleotide and amino acid sequences) using DOTUR [46]. Previous studies showed that the amino acid sequences of AmoA and NosZ similarity around 90% is generally relevant to 97% similarity of 16S rRNA gene [51], [52]. Thus, all these N-cycling gene sequences were classified into OTUs using a 90% amino acid sequence similarity cutoff, and phylogenetic trees were built in ARB using the neighbor-joining method. Sequences of all the samples and genes were also randomly re-sampled to identical sequencing depth (the smallest sequencing effort) using Daisy_chopper (http://www.genomics.ceh.ac.uk/GeneSwytch/Tools.html) to avoid the potential bias caused by sequencing effort difference [53].

The 454-pyrosequencing data were deposited in NCBI SRA under accession number SRA023700.

### Statistical analysis

ANOVA combined with post hoc Tukey B test was used to estimate the difference of archaeal/bacterial *amoA*, *nifH* and *nosZ* genes abundances under different crops based on the quantitative PCR results from the replicated plots. The T-RFLP data from the replicated plots were used to follow the structural changes of soil microbial communities by plant types, and significance tests for these changes were conducted using Analysis of Similarity (ANOSIM) based on Bray–Curtis similarity coefficients. Correspondence analysis (CA) and Canonical correspondence analysis (CCA) were also used to visualize the predominant microbial community changes of archaeal/bacterial *amoA*, *nifH*, *nosZ* and 16S rRNA genes after planting bioenergy crops based on the T-RFLP data. These statistical analyses were done using the free software PAST (http://folk.uio.no/ohammer/past/). Based on our extensive pyrosequencing library, the OTUs/genera that showed monotonic (i.e. continuously increasing or decreasing) trends for each crop treatment over the two year establishment were presumed to be particularly noteworthy in terms of crop impact. The populations with continuously increased or decreased abundance in the two-year period after planting these bioenergy crops were selected using a custom Perl script.

## Results

### Quantification of *nifH*, archaeal *amoA*, bacterial *amoA* and *nosZ* genes

**Figure 1:**
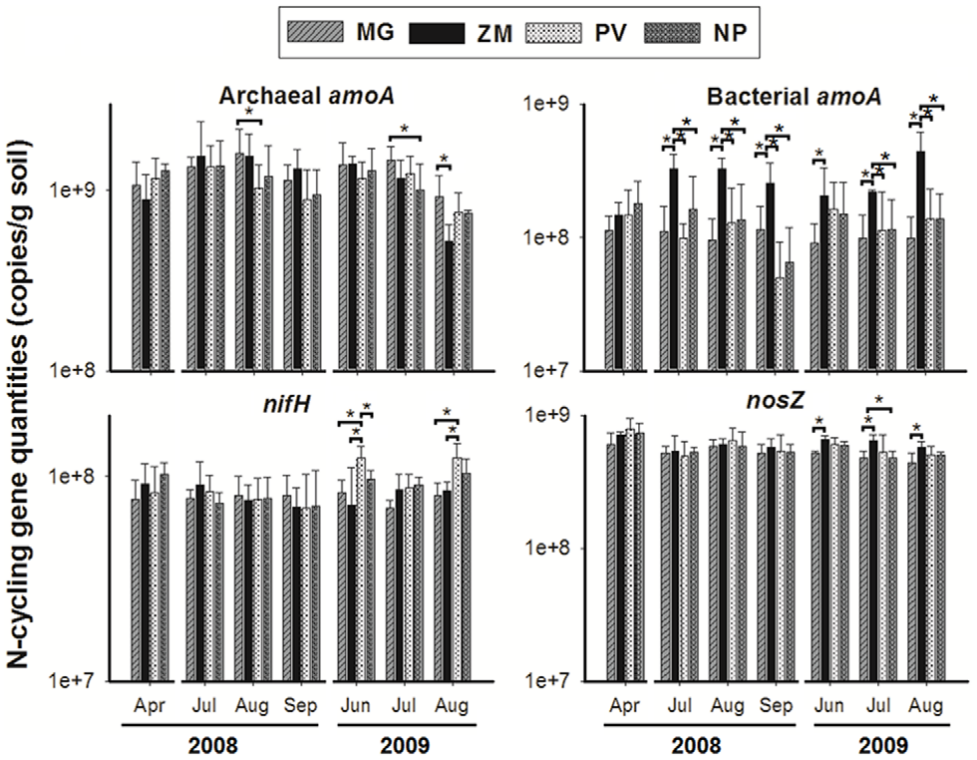
Changes in abundance of *nifH*, archaeal *amoA*, bacterial *amoA* and *nosZ* genes in plots after planting *Miscanthus×giganteus* (MG), *Panicum virgatum* (PV), restored prairie (NP) and *Zea mays* (ZM). The copy number of genes in each gram of dry soil was estimated based on the results of real-time PCR (copy number in each ng DNA) and the average amount of extracted DNA (6.23 µg per dry soil) and assuming DNA extraction efficiency was 30% [40]. The *R^2^* of the standard curve of all these genes was higher than 0.99. The real-time PCR amplification efficiency of *nifH*, archaeal *amoA*, bacterial *amoA* and *nosZ* genes was 1.90±0.01, 1.90±0.06, 1.76±0.01 and 1.82±0.01 respectively. *Represents values that are significantly different (P<0.01).

**Figure 2:**
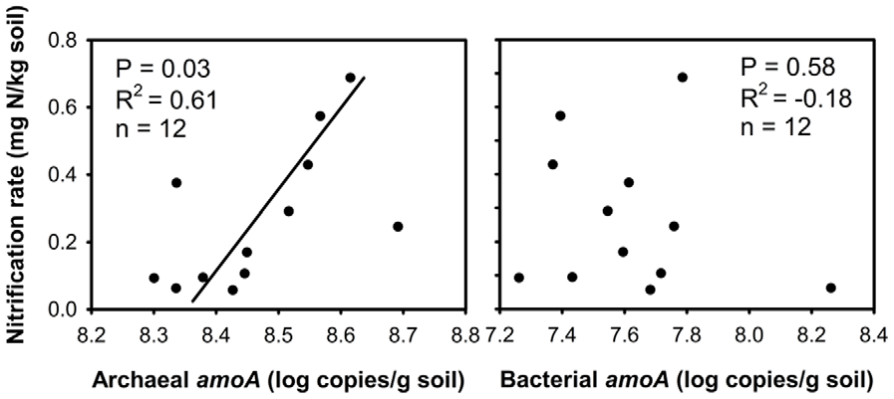
Relationship between the concentration of ammonia-oxidizing archaea/bacteria and nitrification rate. The nitrification rate was determined over the same time as our sample collection in 2009. Nitrification rate was calculated based on the accumulation of nitrate in soil bags incubated in the field (0–10 cm depth) for 15 to 32 days.

Quantities of AOA in all of crops fluctuated over the two growing seasons in a similar pattern (Fig. 1), but the abundance of this gene was always higher in MG than NP and PV. The quantity of bacterial *amoA* genes in ZM significantly increased from 1.47±0.61×10^8^ to 3.26±0.94×10^8^ during the first three months of establishment and thereafter remained higher in ZM than in the other cropping systems. The nitrification rates under these crops were analyzed in the second year by estimating the accumulation of the nitrate in buried soil bags, and linear regression revealed that the nitrification rates were significantly related to the quantities (log) of archaeal *amoA* genes (R^2^ = 0.61, P = 0.03, n = 12), but not to the quantities of bacterial *amoA* genes (Fig. 2). The abundance of *nifH* genes remained stable for all the crops, ranging from 7×10^7^ to 9×10^7^ copies per gram of dry soil (Fig. 1). No significant differences were observed among the different crops in the first year. In the second year, the population sizes of diazotrophs in PV and NP had significantly increased in comparison to the first year (*P* = 0.0001 and 0.0002). The population size of denitrifiers was less variable in comparison to the other N-cycling populations; however, the copy number of *nosZ* increased in ZM during the second year of the study and remained higher than in MG (Fig. 1).

### Structural changes of N-cycling genes and microbial communities after planting of bioenergy crops

**Figure 3:**
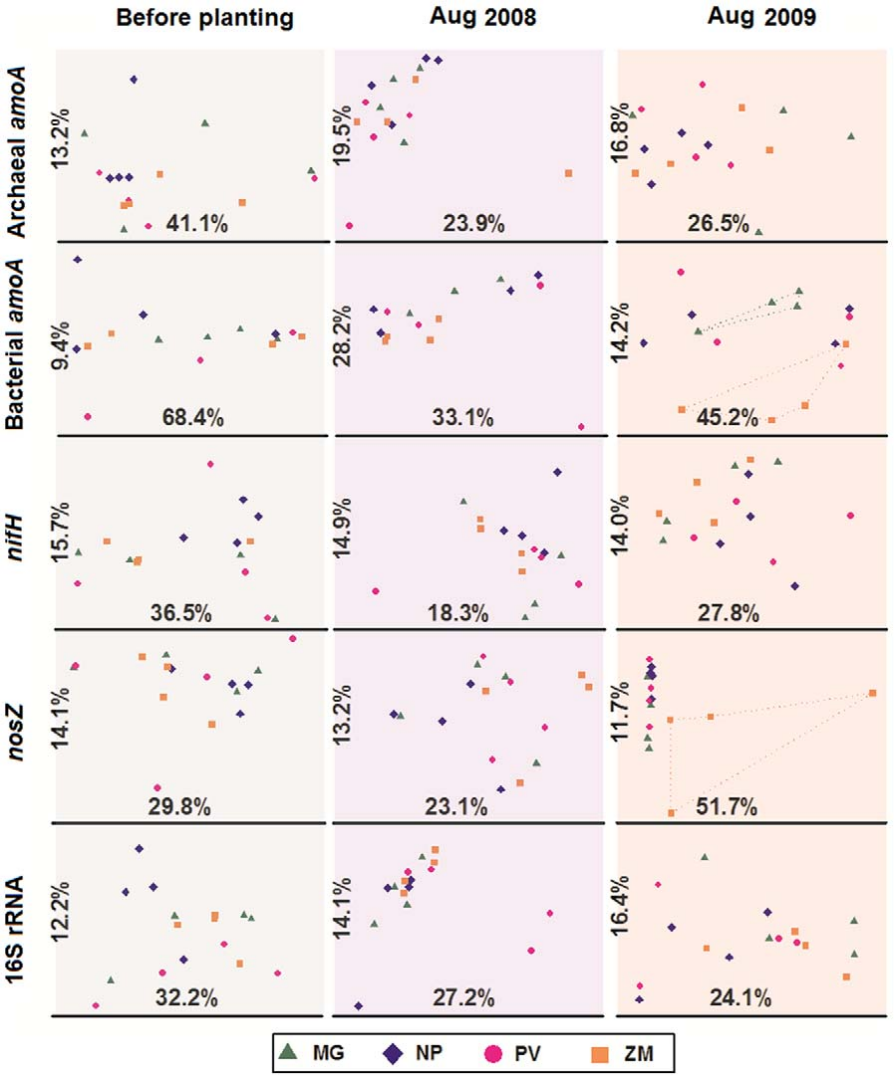
Structural changes of archaeal *amoA*, bacterial *amoA*, *nifH*, *nosZ* and 16S rRNA genes after planting *Miscanthus×giganteus* (MG), *Panicum virgatum* (PV), restored prairie (NP) and *Zea mays* (ZM) revealed by T-RFLP and Correspondence analysis (CA). The number on each axis shows the percentage of total variation explained. The soil samples were collected from four replicate plots for each plant at each time point.

The community structural differentiation of *nifH*, archaeal *amoA*, bacterial *amoA*, *nosZ* and 16S rRNA genes under different bioenergy crops were analyzed by T-RFLP. These analyses used the fully replicated sample set from the randomized block design. Correspondence analysis (Fig. 3) showed that the soil microbial communities in the initial plots did not show any relationship with the crop treatments being applied. During the establishment of these bioenergy crops, the community composition of denitrification bacteria (*nosZ*) under ZM was completely separated (ANOSIM, P<0.05, Table S2) from those under the other crops by the end of the second year along the first-axis, which explained 51.7% of the total variance. None of the other groups showed significant clustering by plant, although the community composition of AOB (bacteria *amoA*) under ZM appeared to be separated from that of MG (ANOSIM, P = 0.17) along the second axis, which explained 14.2% of the total variance. In addition to plant species, the changes of soil microbial communities also could be caused by the variation of environmental conditions. To compare the magnitude of the changes of soil microbial communities related only to plant species, a constrained ordination method was also used. Canonical Correspondence analysis (CCA, Fig. S2) revealed that, at the end of the second year, the microbial communities under ZM were most different from the three cropping systems for bacterial *amoA*, *nosZ*, and 16S rRNA. Archaeal *amoA* was most distinct under MG, followed by ZM, and *nifH* was equally separated under all cropping systems (Fig. S2).

### Diversity of *nifH*, archaeal *amoA*, bacterial *amoA*, *nosZ* and 16S rRNA genes

**Figure 4:**
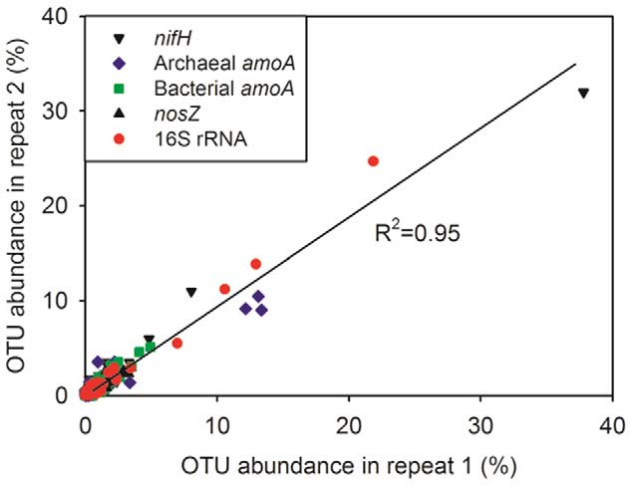
Reproducibility of the pyrosequencing replicates. OTUs of *nifH*, archaeal *amoA*, bacterial *amoA* and *nosZ* genes were classified at a nucleotide similarity cutoff 90%. The 16S rRNA gene sequences were classified to genus level by RDP classifier. One of the samples was duplicated for each gene.

To further understand the composition of microbial community in the field, the *nifH*, archaeal *amoA*, bacterial *amoA*, *nosZ* and 16S rRNA genes were deeply sequenced using the pyrosequencing approach. In total, 143,487 reads were obtained for these genes. The numbers and qualities of these sequences are described in Table 1, Table S3 and Text S1. The reproducibility of the pyrosequencing result was estimated by comparing the observed microbial composition between repeat sequencing runs for all these genes (Fig. 4). Linear regression analysis indicated a high reproducibility (R^2^ = 0.95) of our pyrosequencing.

**Table 1:** Quality of barcoded pyrosequencing reads.

	Number of sequences			
Genes	Correct barcode and primer	Length >350 bp	aValid	Each sample (range)
*nifH*	28,334	27,781	21,111	1,312–2,956
Archaeal *amoA*	16,978	16,226	14,025	697–1,792
Bacterial *amoA*	28,254	27,874	21,817	1,726–2,569
*nosZ*	33,838	28,819	22,590	1,600–2,951
16S rRNA	30,487	30,175	30,101	2,034–3,488
Total	137,891 (96.1%)	130,875 (91.2%)	109,644 (76.4%)	697–3,488

Total number of raw reads was 143,487.

a Valid sequences of *nifH*, archaeal *amoA*, bacterial *amoA* and *nosZ* genes were defined as high quality sequences with correct barcode and primer (at 5′-end), length >350 bp and that did not have frameshifts and chimeric structure. The possible sequencing errors causing frameshifts and chimeras were removed based on the BLASTX result. Sequences with different regions matching the same sequence in the database but with different frame positions were considered to be frameshifts. Sequences that matched two or more different origin sequences were classified as chimeras. Valid sequences of 16S rRNA gene were sequences with correct barcode and primer, length >350 bp and passed the chimeric check program in Greengenes with the Bellerophon method. The sequence numbers for each sample are listed in Table S3.

High diversity of *nifH*, archaeal *amoA*, bacterial *amoA* and *nosZ* genes were observed with 10899, 3187, 3945 and 11242 unique nucleotide sequences and 2286, 2246, 3633 and 4208 unique deduced amino acid sequences respectively (Fig. S3). These sequences were then translated to amino acid sequence according to the BLASTX report. The amino acid sequences of *nifH*, archaeal *amoA*, bacterial *amoA* and *nosZ* genes were classified into 229, 309, 330 and 331 OTUs, respectively, with a similarity cutoff of 90%. After random re-sampling to the same sequencing depth (697 sequences for each sample), the adjusted total number of OTUs for these genes were 217, 303, 319 and 278, respectively (Table S4). Rarefaction analysis of these genes showed that the diversity of archaeal *amoA* gene in ZM2 (second year ZM) and MG1 (first year MG) was markedly lower than the others (Fig. S4). The diversity of *nifH* and *nosZ* genes slightly decreased in ZM2.

The diversity of bacterial and archaeal 16S rRNA genes was much higher than these N-cycling genes. In total, 19,824 unique 16S rRNA gene sequences and 8,989 species (OTUs classified at 97% similarity cutoff) were observed. RDP classification showed that these sequences covered 16 bacterial and 1 archaeal phyla, including 201 genera (Fig. S5). Proteobacteria and Acidobacteria were the most predominant phyla in the soil (>20%). The sequences belonging to Proteobacteria were distributed over 86 different genera, while 94.1% of the sequences in Acidobacteria belonged to Family Gemmatimonadaceae, with GP1 as the most predominant genus (accounted 35.9% of the Acidobacteria).

**Figure 5:**
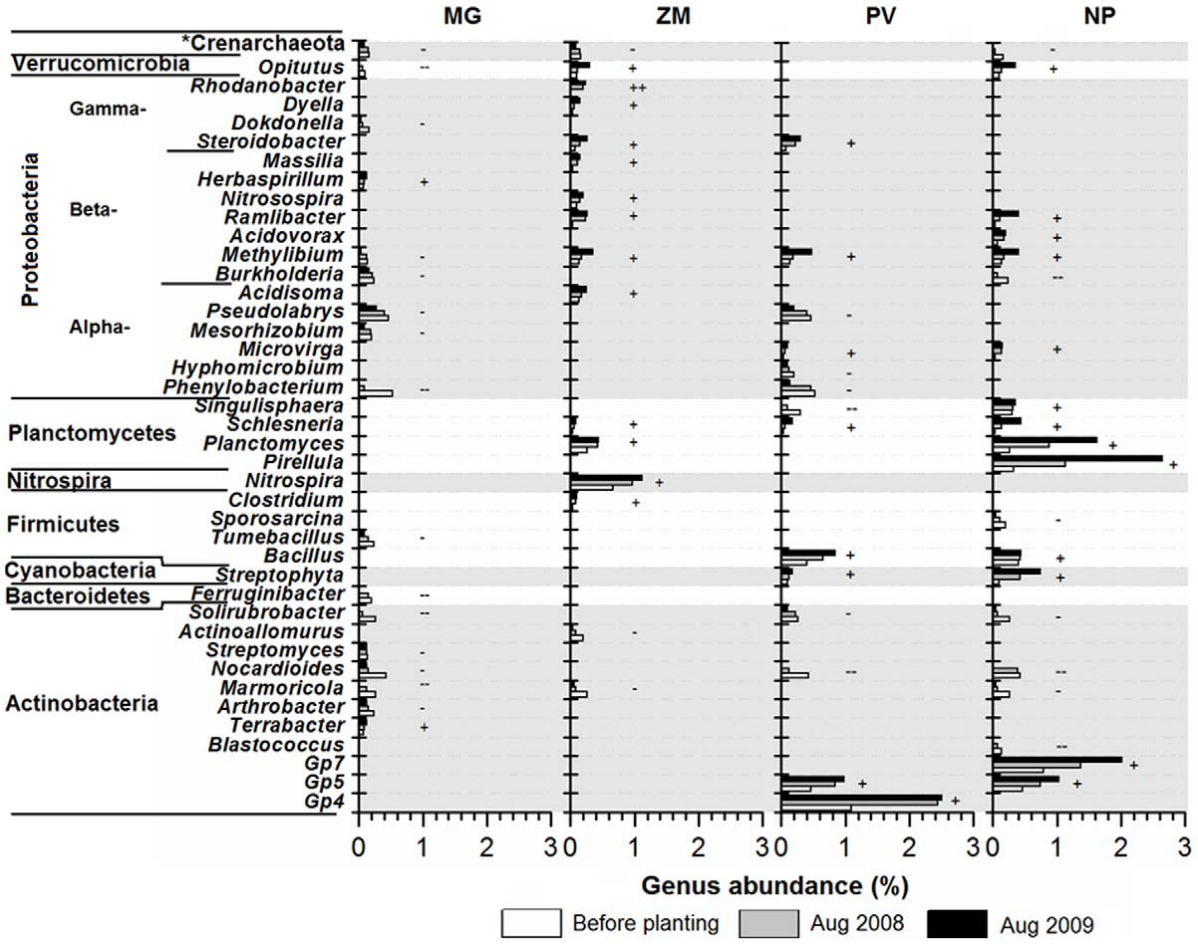
Microbial genera that changed after planting of *Miscanthus×giganteus* (MG), *Panicum virgatum* (PV), restored prairie (NP) and *Zea mays* (ZM). Sequences were classified by RDP Classifier project. −/+ represents the genus continuously decreased/increased after planting the crops; −−/++ represents the genus disappeared/appeared after planting the crops. *sequences belonging to Crenarchaeota, which could not be classified to genus level.

To understand which phylotypes were impacted by vegetation type, the OTUs of *nifH*, archaeal *amoA*, bacterial *amoA*, *nosZ* and 16S rRNA (genus for 16S rRNA) genes that continuously increased or decreased over the two-year establishment of these bioenergy crops were identified (Fig. 5 and Fig. S6, S7, S8, S9). After planting of these bioenergy crops, 27.5%, 15.4%, 22.7% and 14.5% of the total archaeal *amoA*, bacterial *amoA*, *nifH*, and *nosZ* phylotypes, respectively, were found to be continuously increasing or decreasing (Table 2). Details of these continuously changed N-cycling OTUs are described in Text S1 and Figure S6, S7, S8, S9.

**Table 2:** Number of OTUs or genera that changed continuously after planting *Miscanthus×giganteus* (MG), *Panicum virgatum* (PV), restored prairie (NP) and *Zea mays* (ZM).

Genes	MG	PV	ZM	NP	*Total
Archaeal *amoA*	4	16	61	23	85
Bacterial *amoA*	18	18	7	20	51
*nifH*	8	21	14	23	52
*nosZ*	12	18	24	16	48
16S rRNA	17	15	14	21	40

OTUs of N-cycling genes were classified based on a cutoff of 90% amino acid sequences similarity. 16S rRNA genes were classified into genus level by RDP Classifier. Details of the abundance changes of the OTUs or genera are shown in Fig. 5 and S6, S7, S8, S9.

* Total number of changed unique OTUs or genera.

Pyrosequencing of 16S rRNA gene revealed 19.9% of the bacterial genera (39), spanning six phyla, continuously changed after planting of these bioenergy crops (Fig. 5). Only genus *Methylibium* was changed in all the crops, with decreased abundance in MG and increased abundance in the other crops. *Rhodanobacter* only appeared after planting of ZM (7 sequences for both of ZM1 and ZM2), and it was undetectable either in the background soil or in the soil under other crops. Consistent with the changes of *Nitrosospira*-like bacterial *amoA* OTU (see above), the abundance of genus *Nitrosospira* in the 16S rRNA library also increased in ZM. The abundance of genus *Nitrospira*, which is known as a nitrite-oxidizing bacteria, also increased in ZM. Most of the changed genera in MG decreased or even disappeared, except *Terrabacter* and *Herbaspirillum*. All of them were found at low abundance (<1%). Although many genera in Proteobacteria were changed, the total abundance of this most predominant phylum was quite stable under all of the crops (Fig. S5).

## Discussion

In this study, we monitored the structural and quantitative changes of the key genes involved in N-cycling as well as the overall bacterial/archaeal community during two-year establishment of four different bioenergy feedstock crops, and analyzed the shifts of specific soil microbial populations in response to different types of crops. We were able to detect significant changes in the abundance of many of these microbial functional groups within 2 years of initial crop establishment. We also found that traditional row-crop agriculture of maize has a larger impact on the soil N-cycling community than any of the perennial bioenergy feedstock crops (Figs. 1, S2), while the perennial crops were associated with overall community shifts in the phyla Planctomyces, Firmicutes, and Actinobacteria (Fig. 5). Traditional maize cultivation significantly increased the total abundance of ammonia-oxidizing bacteria and denitrifying bacteria (Fig. 1), altered the community composition of denitrifying bacteria (Fig. 3) and decreased the diversity of ammonia-oxidizing archaea, denitrifying bacteria, and diazotrophs (Fig. S4). This may be due to the application of N-fertilizer, which occurred only in ZM plots. Ammonia oxidizers are sensitive to N-fertilizer [24], [27], and these responses were manifested in the increased population size of AOB and the high number of markedly changed AOA species. The nitrification rate was significantly correlated with the quantity of archaeal *amoA*, but not bacterial *amoA*, indicating AOA was the major ammonia-oxidizer.

Deep understanding of the structural shifts of key functional genes can help us to better understand changes in microbial activity in the environment. From the present database, we know that the global diversity of the *nifH*, archaeal *amoA*, bacterial *amoA* and *nosZ* genes, as well as the other functional genes of microorganisms, is high. The traditional approaches (e.g. clone library, DGGE and T-RFLP) used in previous studies may largely underestimate the diversity of microbial communities involved in soil nitrogen cycling. Mounier et al. (2004) revealed that even a large library with 713 clones was insufficient to enumerate the diversity *nosZ* gene in maize rhizosphere, showing the high complexity of N-cycling genes [54]. Thus, high-throughput deep sequencing approaches are essential to improve our knowledge of the diversity of these functional genes. In the present study, using barcoded 454-pyrosequencing approach, we found high diversity (ranging from ∼3100 to ∼11200 unique nucleotide sequences) of *nifH*, archaeal *amoA*, bacterial *amoA* and *nosZ* genes in the soil ecosystem, which far surpasses the diversity of the N-cycling genes observed in previous studies [54], [55], [56], [57], [58], [59], [60], [61]. The rarefaction curves of these genes were close to saturation after sequencing ∼1000 for each sample, indicating that such a sequencing effort is sufficient to elucidate the diversity and structure of the complex soil N-cycling communities. The high similarity between repeat runs of these genes (Fig. 4) demonstrates the high reproducibility and reliability of this barcoded pyrosequencing method. This result also indicates that the variation of pyrosequencing, resulting from random sampling of gene targets during emulsion PCR [62], can be greatly reduced by increasing the sequencing depth and library coverage.

During the establishment of these bioenergy crops, about 15%–30% of N-cycling genes and the detected bacterial/archaeal genera were continuously changed, indicating that a large proportion of soil microbes were affected by the transition to different bioenergy feedstock systems. Most of these phylotypes changed uniquely in one of the crops, indicating that the changes were mainly caused by the particular experimental crop treatment (specific plant species or management, such as fertilization) and not due to the environmental conditions that fluctuated in all treatments (e.g. temperature and moisture). Contrary changes of certain populations in different crops also support this conclusion; for example, the abundance of *Methylibium* (belonging to β-Proteobacteria) decreased in MG but increased in the other crops. The abundance of Bacteroidetes was previously found to be lower in the soil of Miscanthus-dominated grasslands (4%) in comparison to forest soil (6%) [63]. We found that the decrease of Bacteroidetes in MG was mainly due to the disappearance of the genus *Ferruginibacter* (Fig. 5 and S5).

The structure of *nifH* gene was completely separated according to vegetation by the end of the second year (Fig. S2), which suggests that the structure of N-fixation bacterial population was particularly sensitive to plant genotype. Tan et al. (2003) has revealed that the structure of diazotrophs was not only different among rice species, but also changed rapidly with fertilization. The diversity of the *nifH* gene was obviously reduced within 15 days after fertilization [17]. Thus, the decreased diversity of *nifH* in ZM may be also due to the application of fertilizer not the presence of maize. However, the population size of N-fixing bacteria did not change under ZM, indicating N-fertilization may not change the quantity of soil diazotroph [64]. The N-fixing activity was expected to increase in MG [14], [15]. Although the population size of total free-living soil N-fixing bacteria was not significantly increased by growth of MG in the two year period, the abundance of genus *Herbaspirillum* increased. *Herbaspirillum* species are known as endophytic diazotrophs that are enriched by C4-prennial grasses including Miscanthus [65], [66], [67]. Thus, we speculate that the abundance increase of *Herbaspirillum* in the bulk soil was likely due to the root exudates (e.g. organic carbons) released by Miscanthus, which favored the growth of this population. Our results also suggest that Miscanthus may only selectively enhance the activity of specific diazotrophs, not the whole N-fixing microbial community.

The AOA are thought to be more stable and less responsive to environmental differences than AOB, as revealed by previous quantitative studies [6], [68]. However, in the present study we found that, while the population size of AOA was relatively stable, the structure of the AOA community was sensitive to the different cropping systems. The diversity of AOA markedly decreased after planting of maize, with 41 of the AOA phylotypes disappearing (Fig. S6). In contrast, the population size of AOB significantly increased after planting of maize in both the qPCR of bacterial *amoA* and the 16S rRNA pyrosequencing results. In addition to the increase of genus *Nitrosospira* (AOB) [69], the abundance of *Nitrospira* (nitrite-oxidizing bacteria) [70] also increased in N-fertilized maize (Fig. 5). However, the number of changed bacterial *amoA* phylotypes in ZM was much less than the other crops. Therefore, these two different groups of ammonia-oxidizers respond to the N-fertilization in a very different way. The population size of AOB increased immediately in the first growing season, thus, we hypothesize that the increased AOB abundance in maize was likely due not to the growth of maize plants, but to the application of N-fertilizer, which increased the ammonia content in the soil [24], [27]. It has been found that AOB population size increased in seven days after applying of N-fertilizer, and it was still significantly greater than unfertilized soil 8 months after the last application of ammonia [41]. Consistently, we found that the population size of AOB doubled in less than three months and maintained a relatively high level over the two-year study even though measurable NH_4_^+^ in the soil declined over this time period to levels close to that of the unfertilized plots (Fig. 1 and S10). These results indicate the AOB population size can be quickly increased by N-fertilization and can remain for a long period even after the measurable ammonia has been consumed.

The nitrification rate was found to be significantly correlated with the quantity of archaeal *amoA* rather than bacterial *amoA*, indicating AOA rather than AOB may be the major active ammonia-oxidizer in these soils. Contradictory conclusions on the relative importance of AOA and AOB in soil nitrification have been previously reported where nitrification was found to be associated with the changes of archaeal *amoA* abundance or higher archaeal transcriptional activity in some of the soils [58], [71], [72]. In contrast, the nitrification kinetics in the other soils were correlated with the growth of AOB [73], [74]. It has been found that the ammonia affinity of “*Candidatus* Nitrosopumilus maritimus” (a marine AOA) is much higher than AOBs, and its growth may be enhanced by relatively low ammonia concentration [75]. Thus, the contradictory conclusions from these studies may be due to the different soil ammonia concentration used in these experiments [58], [73]. These results hint that AOB may be more active in soils amended with ammonia, while AOA are more active in soils with low ammonia concentration [58]. The nitrification rate outlier (ZM Aug; Fig. 2) had highest population size of AOB, suggesting that the activity of AOB was enhanced by N-fertilization and also supported the above speculation. In support of this speculation, a recent publication shows that recovery of nitrification potential after disruption was dominated by AOB in cropped soils while AOA were responsible RNP in pasture soils [76].

It is known that nitrogen fertilization can change the structure and activity of denitrifying community, and subsequently affect the N_2_O emission [33], [34], [77], [78]. Large amounts (1.3%) of the applied N-fertilizer in maize fields (north Colorado) are converted to N_2_O by the combination of nitrification and denitrification [79]. However, it is still unclear how fertilization changes the microbial community, since most of the previous studies are based on the already established fields [7], [29], [33]. Our study revealed that the structure of denitrifying bacteria in maize soil was significantly differentiated from the other crops at the second year (Fig. 3). However, the population size of denitrifiers was relatively stable in all the crops in comparison to other N-cycling microbial communities, which only slightly increased in maize at the second year. The high stability of denitrifying population abundance could be explained by the high diversity and functional redundancy of denitrification community [30], [80].

In conclusion, our two-year study of transitional agriculture shows that specific N-transforming microbial communities develop in the soil in response to different bioenergy crops. Each N-cycling microbial group responded in a different way after planting with different bioenergy crops. In general, planting of maize has a larger impact on the soil N-cycling community than the other bioenergy crops. Our results also indicate that application of N-fertilizer may not only cause short-term environmental problems, e.g. water contamination, but also can have long-term influence on the global biogeochemical cycles through changing the soil microbial community structure and abundance. Since soil types and other environmental factors may also impact the N-cycling microbial community, the universality of our findings needs to be confirmed by additional study at different sites.

## Supporting Information

Figure S1 Number of OTUs obtained by two different processing methods: QIIME (denoised) and RDP pyrosequencing pipeline (non-denoised).
(TIF)

Figure S2 Structural changes of archaeal *amoA*, bacterial *amoA*, *nifH*, *nosZ* and 16S rRNA genes after planting *Miscanthus×giganteus* (MG), *Panicum virgatum* (PV), restored prairie (NP) and *Zea mays* (ZM) revealed by T-RFLP and Canonical correspondence analysis (CCA). The number on each axis shows the explained total variation. The soil samples were collected from four replicated plots for each plant at each time point. * Correspondence analysis was used for the samples collected before planting bioenergy crops.
(TIF)

Figure S3 OTU classification of valid sequences at different distance levels based on nucleotide and deduced amino acid sequences.
(TIF)

Figure S4 Rarefaction analysis of the diversities of *nifH*, archaeal *amoA*, bacterial *amoA*, *nosZ* and 16S rRNA genes in the soil underneath different bioenergy crops. The OTUs of *nifH*, archaeal *amoA*, bacterial *amoA* and *nosZ* genes were classified at 90% similarity cutoff based on amino acid sequences, and 16S rRNA gene was classified at 97% similarity cutoff on nucleotide sequences. BG0 represents the samples collected before planting bioenergy crops. MG, PV, NP, and ZM represent *Miscanthus×giganteus*, *Panicum virgatum*, restored prairie and *Zea mays* respectively. 1 and 2 represent samples collected in the first and second growing seasons.
(TIF)

Figure S5 Phylum level microbial community composition in the soil under different plants before and for two years after transition to bioenergy cropping. * represent significantly changed phylum. MG, *Miscanthus×giganteus*; PV, *Panicum virgatum*; NP, restored prairie; ZM, *Zea mays*.
(TIF)

Figure S6 (a) Phylogenetic tree of and (b) abundance of archaeal *amoA* OTUs that continuously changed after planting *Miscanthus×giganteus* (MG), *Panicum virgatum* (PV), restored prairie (NP) and *Zea mays* (ZM). OTUs were classified based on a cutoff of 90% amino acid sequence similarity.
(TIF)

Figure S7 (a) Phylogenetic tree of and (b) abundance of bacterial *amoA* OTUs that continuously changed after planting *Miscanthus×giganteus* (MG), *Panicum virgatum* (PV), restored prairie (NP) and *Zea mays* (ZM). OTUs were classified based on a cutoff of 90% amino acid sequence similarity.
(TIF)

Figure S8 (a) Phylogenetic tree of and (b) abundance of *nifH* OTUs that continuously changed after planting *Miscanthus×giganteus* (MG), *Panicum virgatum* (PV), restored prairie (NP) and *Zea mays* (ZM). OTUs were classified based on a cutoff of 90% amino acid sequence similarity.
(TIF)

Figure S9 (a) Phylogenetic tree of and (b) abundance of *nosZ* OTUs that continuously changed after planting *Miscanthus×giganteus* (MG), *Panicum virgatum* (PV), restored prairie (NP) and *Zea mays* (ZM). OTUs of were classified based on a cutoff of 90% amino acid sequence similarity.
(TIF)

Figure S10 Nitrate and ammonia concentration in bulk soil. Soil samples for chemical and microbiological analysis were collected in the same week for each time point, except Sep 2008 when the nitrate and ammonia concentrations were not measured. MG, *Miscanthus×giganteus*; PV, *Panicum virgatum*; NP, restored prairie; ZM, *Zea mays*. These data were measured over the same period of our sample collection by the Biogeochemistry laboratory (C. Smith and M. David).
(TIF)

Figure S11 Pyrosequencing read length based on the raw sequence reads.
(TIF)

Table S1 Primers and annealing temperature for *nifH*, archaeal *amoA*, bacterial *amoA, nosZ* and 16S rRNA genes.
(DOC)

Table S2 Comparsion of the microbial community structures between different crops.
(DOC)

Table S3 Number of valid sequences for each gene in each sample.
(DOCX)

Table S4 Number of OTUs observed after random re-sampling to the identical sequencing depth (697 sequences/sample.
(DOCX)

Text S1 (DOC)
