# Supplementary figures and images for: Changes in N-Transforming Archaea and Bacteria in Soil during the Establishment of Bioenergy Crops
Source: PLoS One. 2011 Sep 14;6(9):e24750. doi: 10.1371/journal.pone.0024750 (PMC3173469; doi:10.1371/journal.pone.0024750)

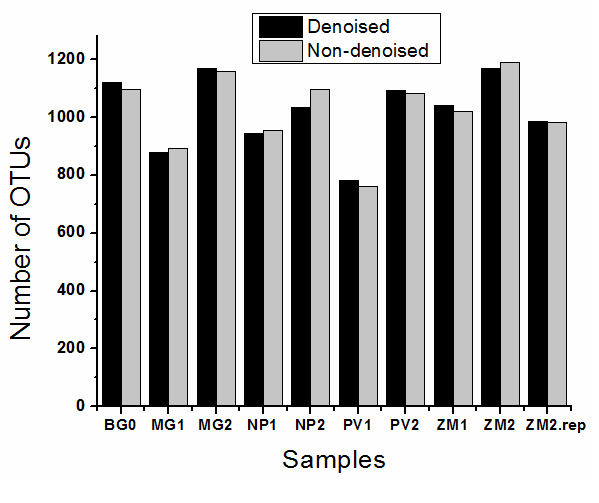

Supplement: Figure S1 — Number of OTUs obtained by two different processing methods: QIIME (denoised) and RDP pyrosequencing pipeline (non-denoised). (TIF) [file pone.0024750.s001.tif]

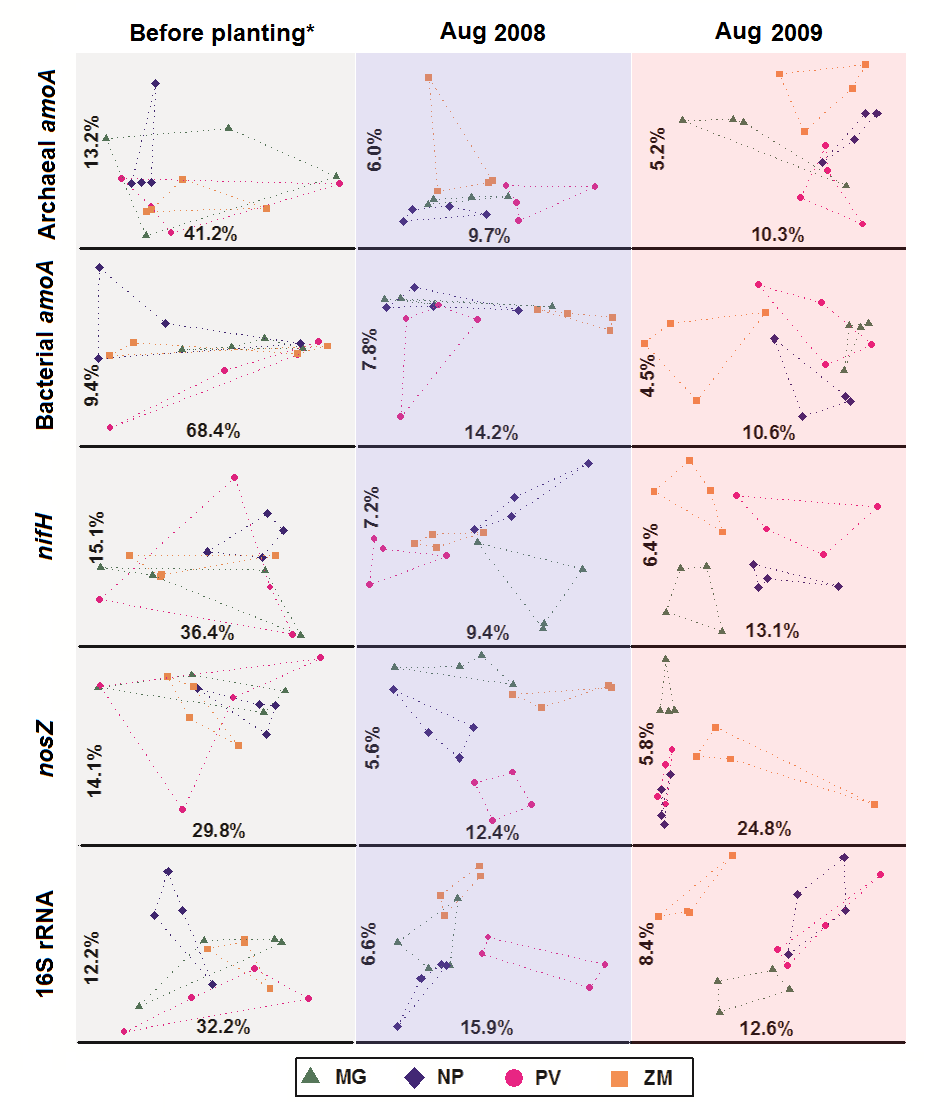

Supplement: Figure S2 — Structural changes of archaeal amoA, bacterial amoA, nifH, nosZ and 16S rRNA genes after planting Miscanthus×giganteus (MG), Panicum virgatum (PV), restored prairie (NP) and Zea mays (ZM) revealed by T-RFLP and Canonical correspondence analysis (CCA). The number on each axis shows the explained total variation. The soil samples were collected from four replicated plots for each plant at each time point. * Correspondence analysis was used for the samples collected before planting bioenergy crops. (TIF) [file pone.0024750.s002.tif]

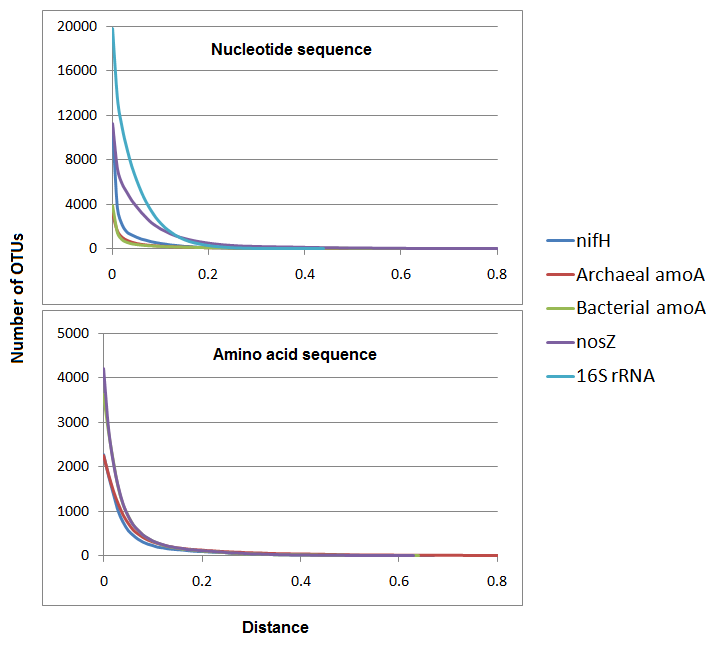

Supplement: Figure S3 — OTU classification of valid sequences at different distance levels based on nucleotide and deduced amino acid sequences. (TIF) [file pone.0024750.s003.tif]

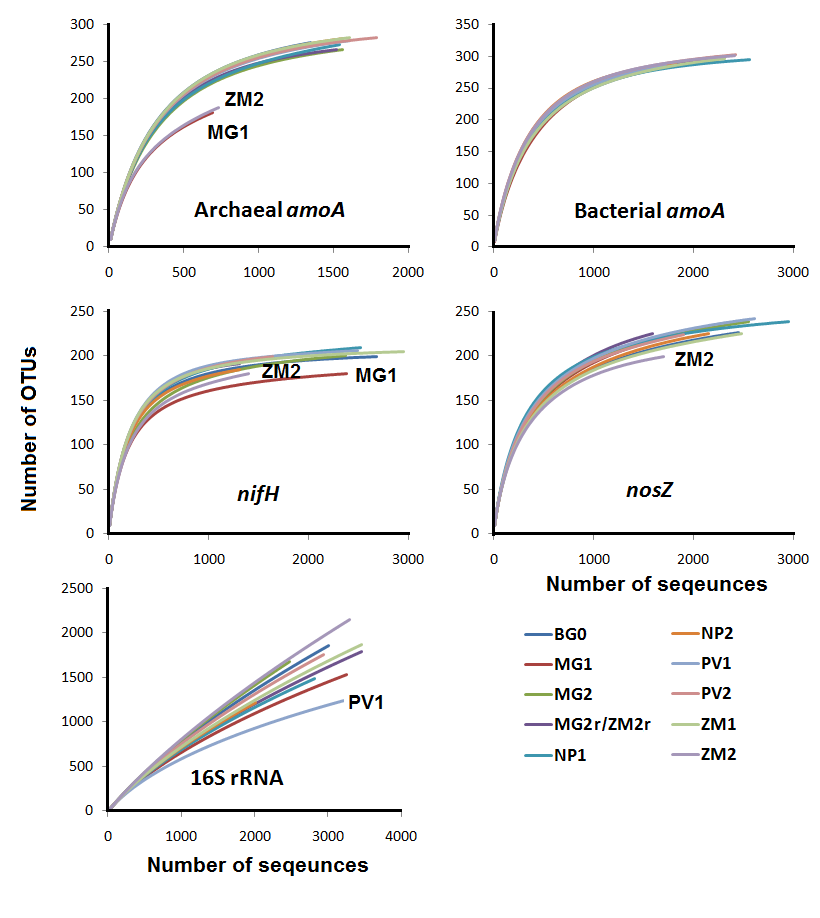

Supplement: Figure S4 — Rarefaction analysis of the diversities of nifH, archaeal amoA, bacterial amoA, nosZ and 16S rRNA genes in the soil underneath different bioenergy crops. The OTUs of nifH, archaeal amoA, bacterial amoA and nosZ genes were classified at 90% similarity cutoff based on amino acid sequences, and 16S rRNA gene was classified at 97% similarity cutoff on nucleotide sequences. BG0 represents the samples collected before planting bioenergy crops. MG, PV, NP, and ZM represent Miscanthus×giganteus, Panicum virgatum, restored prairie and Zea mays respectively. 1 and 2 represent samples collected in the first and second growing seasons. (TIF) [file pone.0024750.s004.tif]

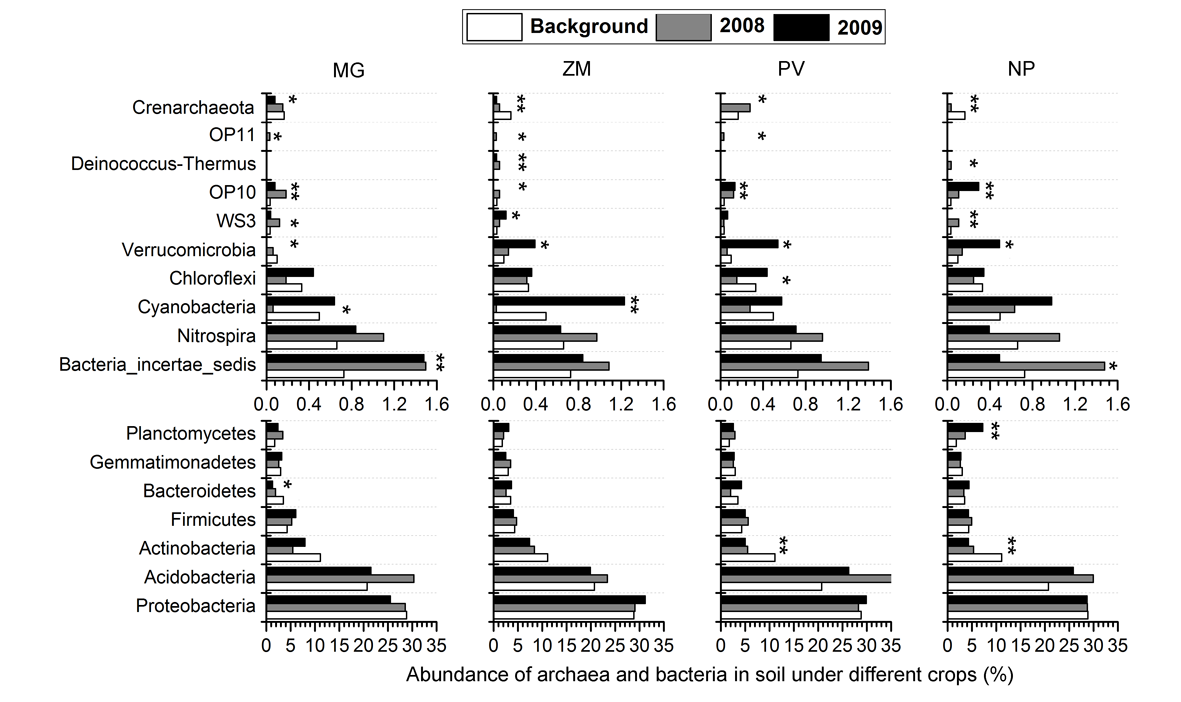

Supplement: Figure S5 — Phylum level microbial community composition in the soil under different plants before and for two years after transition to bioenergy cropping. * represent significantly changed phylum. MG, Miscanthus×giganteus; PV, Panicum virgatum; NP, restored prairie; ZM, Zea mays. (TIF) [file pone.0024750.s005.tif]

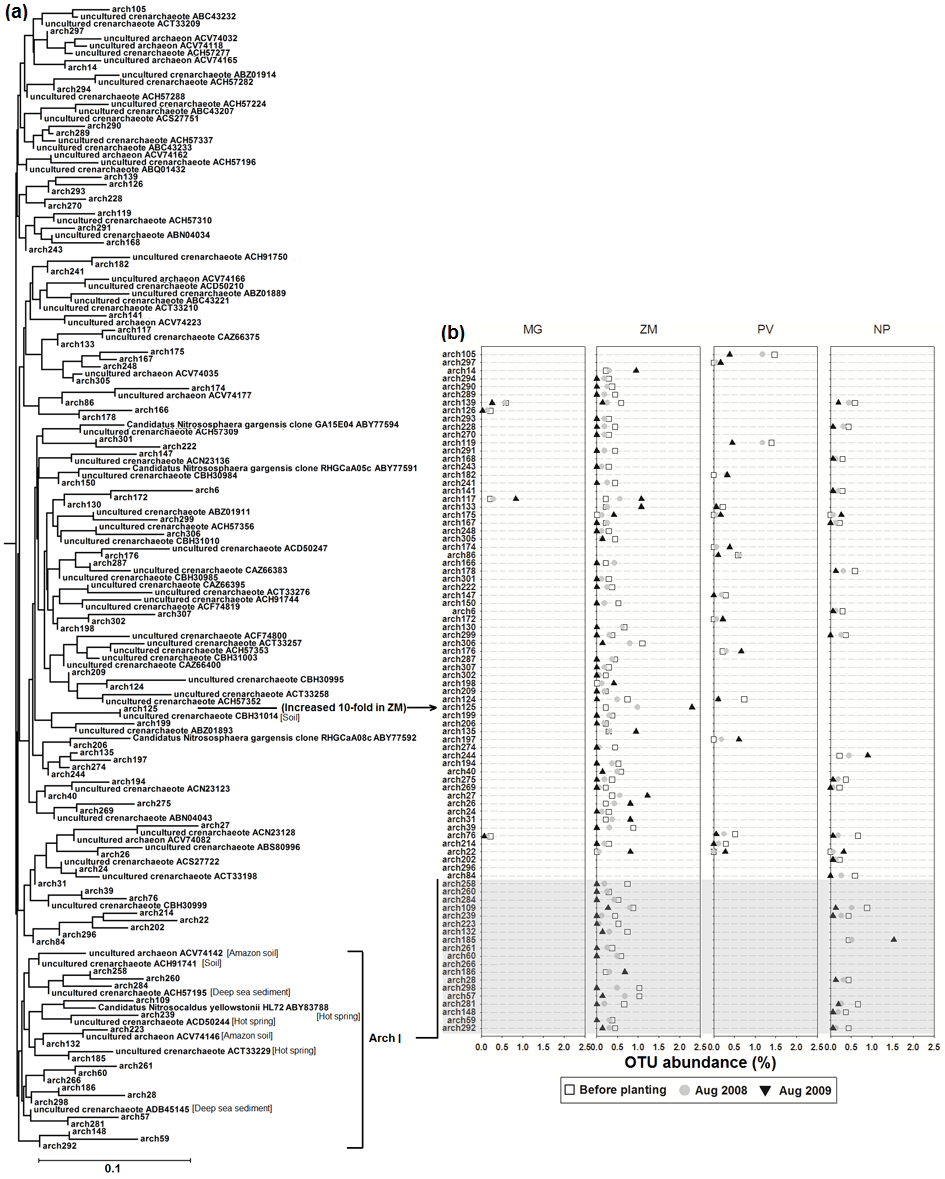

Supplement: Figure S6 — (a) Phylogenetic tree of and (b) abundance of archaeal amoA OTUs that continuously changed after planting Miscanthus×giganteus (MG), Panicum virgatum (PV), restored prairie (NP) and Zea mays (ZM). OTUs were classified based on a cutoff of 90% amino acid sequence similarity. (TIF) [file pone.0024750.s006.tif]

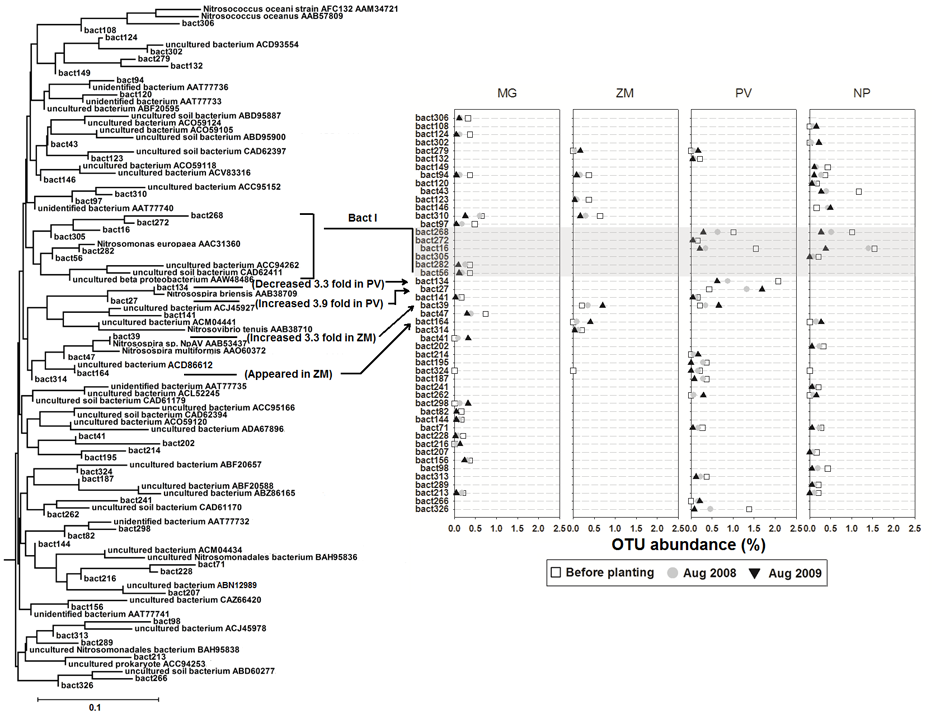

Supplement: Figure S7 — (a) Phylogenetic tree of and (b) abundance of bacterial amoA OTUs that continuously changed after planting Miscanthus×giganteus (MG), Panicum virgatum (PV), restored prairie (NP) and Zea mays (ZM). OTUs were classified based on a cutoff of 90% amino acid sequence similarity. (TIF) [file pone.0024750.s007.tif]

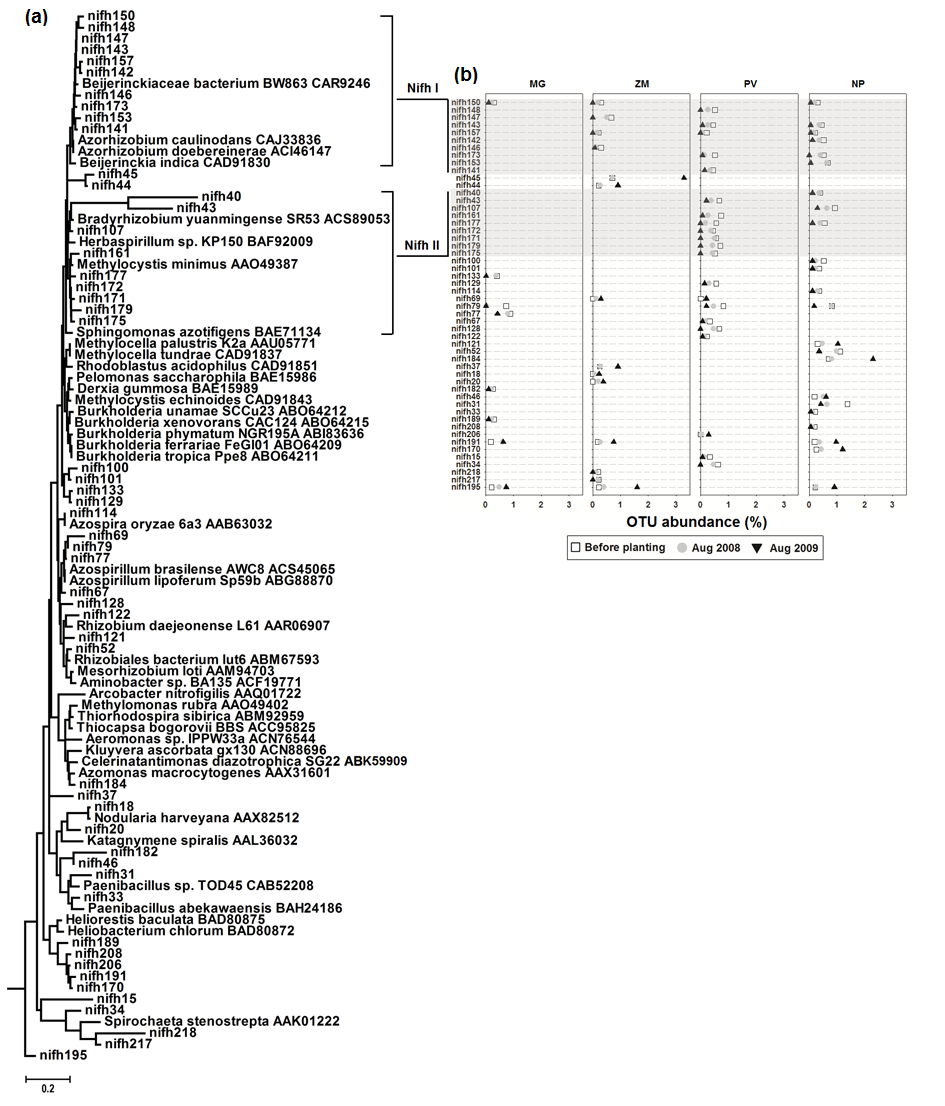

Supplement: Figure S8 — (a) Phylogenetic tree of and (b) abundance of nifH OTUs that continuously changed after planting Miscanthus×giganteus (MG), Panicum virgatum (PV), restored prairie (NP) and Zea mays (ZM). OTUs were classified based on a cutoff of 90% amino acid sequence similarity. (TIF) [file pone.0024750.s008.tif]

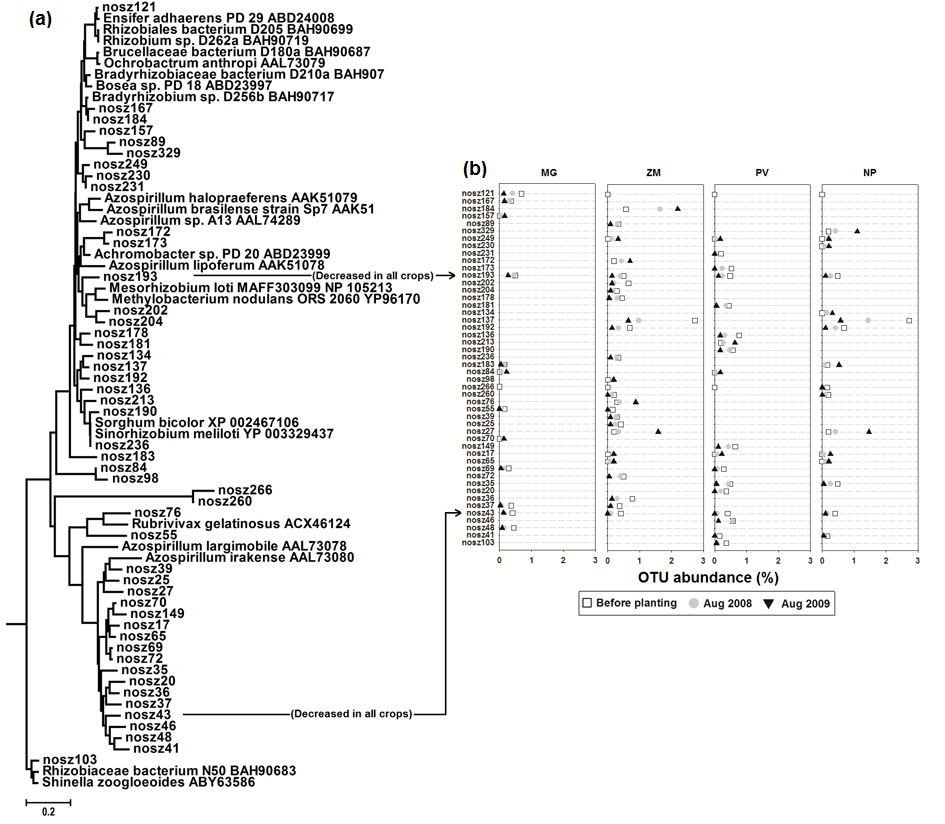

Supplement: Figure S9 — (a) Phylogenetic tree of and (b) abundance of nosZ OTUs that continuously changed after planting Miscanthus×giganteus (MG), Panicum virgatum (PV), restored prairie (NP) and Zea mays (ZM). OTUs of were classified based on a cutoff of 90% amino acid sequence similarity. (TIF) [file pone.0024750.s009.tif]

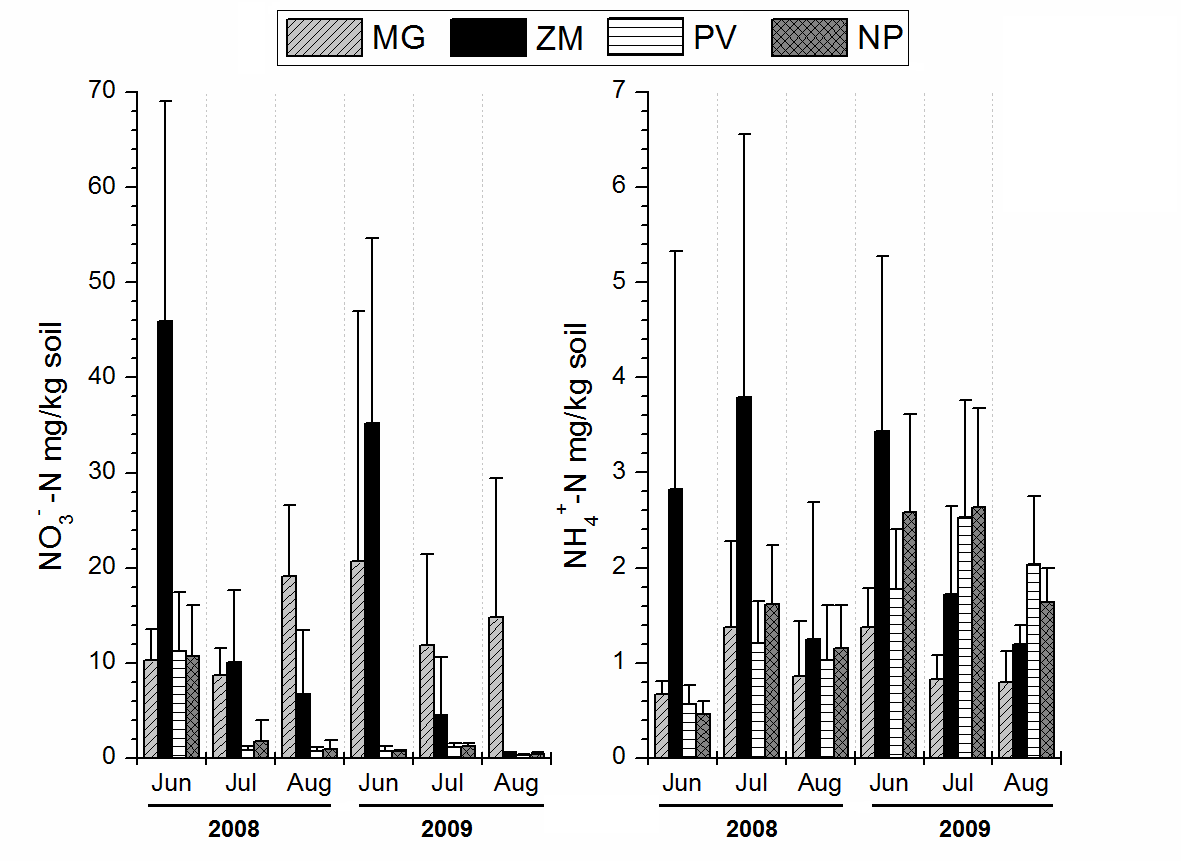

Supplement: Figure S10 — Nitrate and ammonia concentration in bulk soil. Soil samples for chemical and microbiological analysis were collected in the same week for each time point, except Sep 2008 when the nitrate and ammonia concentrations were not measured. MG, Miscanthus×giganteus; PV, Panicum virgatum; NP, restored prairie; ZM, Zea mays. These data were measured over the same period of our sample collection by the Biogeochemistry laboratory (C. Smith and M. David). (TIF) [file pone.0024750.s010.tif]

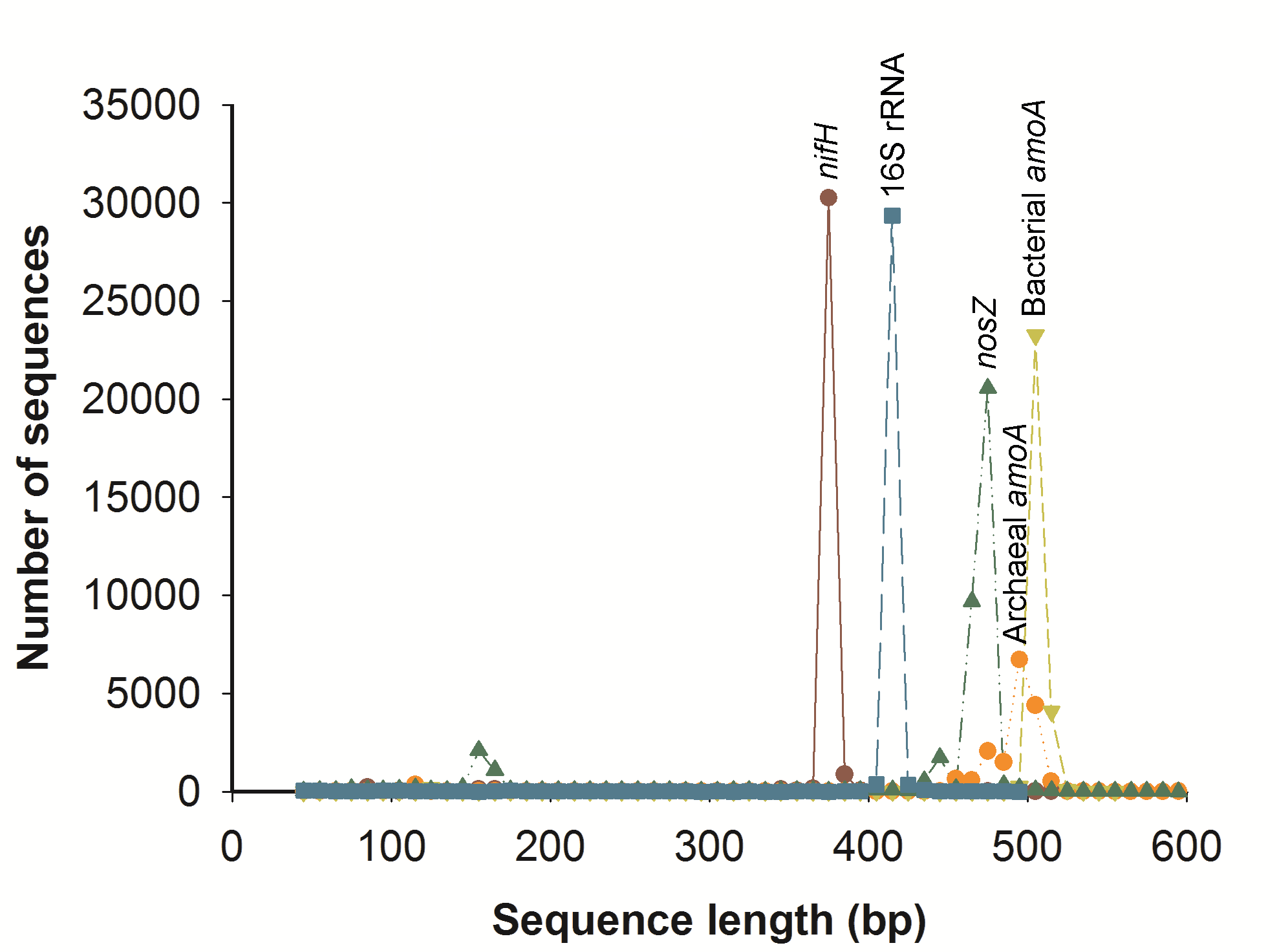

Supplement: Figure S11 — Pyrosequencing read length based on the raw sequence reads. (TIF) [file pone.0024750.s011.tif]
