# Supplementary material for: Changes in N-Transforming Archaea and Bacteria in Soil during the Establishment of Bioenergy Crops
Source: PLoS One. 2011 Sep 14;6(9):e24750. doi: 10.1371/journal.pone.0024750 (PMC3173469; doi:10.1371/journal.pone.0024750)
Supplement: Table S1 — Primers and annealing temperature for nifH, archaeal amoA, bacterial amoA, nosZ and 16S rRNA genes. (DOC) [file pone.0024750.s012.doc]

Table S1. Primers and annealing temperature for *nifH*, archaeal *amoA*, bacterial *amoA, nosZ* and 16S rRNA genes.

|  |  |  |  | Annealing temperature (^o^C) | |  |
| --- | --- | --- | --- | --- | --- | --- |
| Genes | Primers | Sequences (5' to 3'-end) | Product length (~bp) | Real-time PCR | 454 sequencing | References |
| *nifH* | PolF | TGCGAYCCSAARGCBGACTC | 360 | 54 | 54 | [1] |
|  | PolR | ATSGCCATCATYTCRCCGGA |  |  |  |  |
| Archaeal *amoA* | Arch-amoAF | STA ATGGTCTGGCTTAGACG | 635 | 56 | 60 | [2] |
|  | Arch-amoAR | GCGGCCATCCATCTGTATG |  |  |  |  |
| Bacterial *amoA* | amoA-1F | GGGGTTTCTACTGGTGGT | 490 | 56 | 56 | [3] |
|  | amoA-2R | CCCCTCKGSAAAGCCTTCTTC |  |  |  |  |
| *nosZ* | nosZ-F | CGYTGTTCMTCGACAGCCAG | 450 | 56 | 60 | [4] |
|  | nosZ-R | CGSACCTTSTTGCCSTYGCG |  |  |  |  |
| 16S rRNA | U519F | CAGCMGCCGCGGTAATWC | 407 | - | 60 | [5] |
|  | U926R | CCGTCAATTCMTTTRAGTT |  |  |  |  |

Different annealing temperatures were used for real-time PCR and 454-sequencing for some of the genes, because the DNA polymerase kit and PCR mixture content were different.
