# Supplementary material for: Changes in N-Transforming Archaea and Bacteria in Soil during the Establishment of Bioenergy Crops
Source: PLoS One. 2011 Sep 14;6(9):e24750. doi: 10.1371/journal.pone.0024750 (PMC3173469; doi:10.1371/journal.pone.0024750)
Supplement: Table S2 — Comparsion of the microbial community structures between different crops. (DOC) [file pone.0024750.s013.doc]

Table S2. Comparsion of the microbial community structures between different crops.

| Genes | Time | MG vs NP | MG vs PV | MG vs ZM | NP vs PV | NP vs ZM | PV vs ZM |
| --- | --- | --- | --- | --- | --- | --- | --- |
| Archaeal amoA | Before planting | -0.02^a^ | -0.25 | -0.25 | -0.14 | 0.01 | -0.11 |
|  | 2008 | -0.32 | -0.07 | -0.19 | 0.06 | -0.10 | -0.08 |
|  | 2009 | 0.03 | -0.08 | -0.08 | -0.01 | -0.10 | 0.04 |
| Bacterial amoA | Before planting | 0.25 | -0.14 | 0.04 | 0.05 | -0.13 | -0.15 |
|  | 2008 | -0.16 | -0.04 | 0.51 | -0.15 | 0.31 | 0.29 |
|  | 2009 | 0.09 | 0.04 | 0.24 | -0.16 | -0.03 | -0.01 |
| nifH | Before planting | 0.33 | -0.19 | -0.10 | 0.09 | 0.39 | -0.05 |
|  | 2008 | -0.01 | -0.07 | 0.03 | 0.10 | 0.00 | 0.10 |
|  | 2009 | 0.07 | 0.03 | 0.00 | -0.14 | 0.14 | 0.14 |
| nosZ | Before planting | 0.01 | -0.19 | 0.01 | 0.00 | 0.15 | -0.01 |
|  | 2008 | -0.16 | 0.00 | 0.03 | 0.03 | 0.19 | -0.02 |
|  | 2009 | 0.16 | 0.13 | ***0.31*^b^*** | -0.07 | ***0.33**** | ***0.23**** |
| 16S rRNA | Before planting | 0.10 | -0.17 | 0.08 | 0.04 | 0.23 | -0.01 |
|  | 2008 | -0.02 | 0.08 | -0.03 | 0.23 | 0.16 | 0.21 |
|  | 2009 | 0.16 | -0.11 | 0.21 | -0.23 | 0.35 | 0.16 |

^a^R indicates the degree of separation between two communities, with score 0 indicating no separation.

^b^P-value: *P<0.05.
