# Supplementary material for: Changes in N-Transforming Archaea and Bacteria in Soil during the Establishment of Bioenergy Crops
Source: PLoS One. 2011 Sep 14;6(9):e24750. doi: 10.1371/journal.pone.0024750 (PMC3173469; doi:10.1371/journal.pone.0024750)
Supplement: Table S3 — Number of valid sequences for each gene in each sample. (DOCX) [file pone.0024750.s014.docx]

Table S3. Number of valid sequences for each gene in each sample.

| Samples | *nifH* | Archaeal *amoA* | Bacterial *amoA* | *nosZ* | 16S rRNA |
| --- | --- | --- | --- | --- | --- |
| BG0 | 2688 | 1358 | 1880 | 2453 | 3021 |
| MG1 | 2386 | 697 | 2279 | 2162 | 3263 |
| MG2 | 2374 | 1570 | 2248 | 2556 | 2491 |
| MG2_rep | 1319 | 1528 | 2133 | 1600 | - |
| NP1 | 2529 | 1541 | 2569 | 2951 | 2835 |
| NP2 | 1312 | 1564 | 1822 | 2151 | 2034 |
| PV1 | 2495 | 1617 | 1726 | 2619 | 3230 |
| PV2 | 1650 | 1792 | 2422 | 1904 | 2949 |
| ZM1 | 2956 | 1619 | 2318 | 2487 | 3488 |
| ZM2 | 1402 | 739 | 2420 | 1707 | 3317 |
| ZM2_rep | - | - | - | - | 3473 |
