# Supplementary material for: Changes in N-Transforming Archaea and Bacteria in Soil during the Establishment of Bioenergy Crops
Source: PLoS One. 2011 Sep 14;6(9):e24750. doi: 10.1371/journal.pone.0024750 (PMC3173469; doi:10.1371/journal.pone.0024750)
Supplement: Table S4 — Number of OTUs observed after random re-sampling to the identical sequencing depth (697 sequences/sample. (DOCX) [file pone.0024750.s015.docx]

Table S4. Number of OTUs observed after random re-sampling to the identical sequencing depth (697 sequences/sample).

| Genes | BG0 | MG1 | MG2 | MG2_rep | NP1 | NP2 | PV1 | PV2 | ZM1 | ZM2 | ZM2_rep | *Total |
| --- | --- | --- | --- | --- | --- | --- | --- | --- | --- | --- | --- | --- |
| 16S rRNA | 545 | 477 | 551 | - | 485 | 488 | 433 | 535 | 510 | 562 | 492 | 3117 |
| Archaeal *amoA* | 221 | 182 | 213 | 210 | 209 | 226 | 207 | 222 | 221 | 172 | - | 303 |
| Bacterial *amoA* | 203 | 204 | 200 | 215 | 218 | 221 | 220 | 220 | 220 | 230 | - | 319 |
| *nifH* | 163 | 139 | 155 | 162 | 159 | 162 | 171 | 167 | 177 | 150 | - | 217 |
| *nosZ* | 152 | 164 | 174 | 166 | 165 | 157 | 171 | 161 | 158 | 152 | - | 278 |

Sequences of all the samples and genes were randomly re-sampled to identical sequencing depth (697 sequences/sample) using Daisy_chopper. OTUs of *nifH*, archaeal *amoA*, bacterial *amoA* and *nosZ* genes were classified at a amino acid similarity cutoff 90%. OTUs of 16S rRNA gene were classified at a similarity cutoff 97%.

*Total number of unique OTUs.
