# Supplementary material for: Changes in N-Transforming Archaea and Bacteria in Soil during the Establishment of Bioenergy Crops
Source: PLoS One. 2011 Sep 14;6(9):e24750. doi: 10.1371/journal.pone.0024750 (PMC3173469; doi:10.1371/journal.pone.0024750)
Supplement: Text S1 — (DOC) [file pone.0024750.s016.doc]

**Pyrosequencing quality**

In total, 143,487 reads were obtained for the 16S rRNA and four N-cycling functional genes by pyrosequencing (Table 1). Correct barcodes and primers were found in 96.1% of these raw reads. Most of the bacterial *amoA*, archaeal *amoA* and *nosZ* gene sequences were around 500bp length, *nifH* gene sequences were around 360bp, 16S rRNA gene sequences were around 400bp (Fig. S11). 91.2% of these sequences with correct barcode and primer were longer than 350bp. BLASTX analysis of these N-cycling gene sequences showed that chimeric structure and frameshifts, potentially caused by sequencing errors, existed in many of these sequences (Table 1). After screening these N-cycling gene sequences with our strict criteria (described in methods), 79,543 valid sequences were obtained, which accounted 72.1% of their raw reads. The 16S rRNA gene sequences were screened by NAST, and 30,101 sequences were obtained for the following analysis, which accounted for 98.7% of their raw reads. The number of valid sequences for each sample ranged from 697 to 3488. Although the mole amounts of the sequenced PCR products for these N-cycling genes were the same, the number of archaeal *amoA* sequences was much lower than the other genes, with valid sequences approximately 1/3 lower than the others.

The variation of this barcoded pyrosequencing method was estimated by comparing the sequences obtained from two technical replicates for all of these genes (second year MG for N-cycling genes, labeled as MG2 and MG2 rep; second year ZM for 16S rRNA genes, labeled as ZM2 and ZM2 rep). The abundance of each OTU or genera in these technical replicates were significantly correlated (R^2^=0.95, P<0.001, n=2879; Fig. 4), demonstrating the high reproducibility of the method. Although the numbers of sequences were quite different between the technical replicates of *nifH* and *nosZ* genes, the reproducibility of *nifH* gene was almost not affected (R^2^=0.97); however, the correlation coefficient of *nosZ* repetitions (0.75) was lower than the other genes.

**Continuously changed N-cycling OTUs after planting bioenergy crops**

Only four AOA OTUs were changed in MG, while more than half of these changed AOA OTUs (44) were found to be continuously altered in ZM plots (Fig. S6). Most of the OTUs in group Arch I, which shared relative high similarities with AOAs from diverse habitats (e.g. hot springs, soils and deep sea sediments), disappeared after planting ZM but none of them changed in PV and MG (Fig. S6). Abundance of arch125, which shared 100% similarity with many uncultured archaea (from sediments or soil) in NCBI, was increased from 0.22% to 2.3% after planting ZM, and by the end of the second year it became the most abundant OTU in ZM. Similar numbers of bacterial *amoA* OTUs (18 to 20) were found to be changed —mostly decreased — after planting the crops without fertilization (MG, PV and NP), while fewer of these OTUs (7) changed in ZM (Fig. S7). For example, the abundance of OTUs in group Bact I, which were related to *Nitrosomonas europaea*, were decreased after planting MG, PV and NP, but none changed in ZM. In ZM, only the abundance of bact39 and bact164, which shared 100% similarity with *Nitrosospira* sp. NpAV (AAB53437) and uncultured bacterium (ACD86612) respectively, was increased. Some of the closely related bacterial *amoA* OTUs responded differently to planting of the same plant, e.g. abundance of bact27 was increased 3.9-fold (from 0.43 to 1.69) while bact134 was decreased 3.3-fold (from 2.07 to 0.62) in PV, both of these OTUs are close to *Nitrosopira briensis* (Fig. S7). Only eight *nifH* phylotyps with abundance <1% were changed in MG, which is much lower than the other crops (ranging from 14 to 23). Part of these diazotrophs was selectively influenced by these crops, e.g. the abundance of OTUs in group Nifh II which belonging to Proteobacteria decreased in PV and NP, but not in MG and ZM (Fig. S8). Different *nosZ* phylotypes were affected by each crop, except nosz43 and nosz193, which were decreased in all crops (Fig. S9).

**References**

1. Poly F, Ranjard L, Nazaret S, Gourbiere F, Monrozier LJ (2001) Comparison of nifH gene pools in soils and soil microenvironments with contrasting properties. Appl Environ Microbiol 67: 2255-2262.

2. Francis CA, Roberts KJ, Beman JM, Santoro AE, Oakley BB (2005) Ubiquity and diversity of ammonia-oxidizing archaea in water columns and sediments of the ocean. Proc Natl Acad Sci U S A 102: 14683-14688.

3. Rotthauwe JH, Witzel KP, Liesack W (1997) The ammonia monooxygenase structural gene amoA as a functional marker: molecular fine-scale analysis of natural ammonia-oxidizing populations. Appl Environ Microbiol 63: 4704-4712.

4. Ruiz-Rueda O, Hallin S, Baneras L (2009) Structure and function of denitrifying and nitrifying bacterial communities in relation to the plant species in a constructed wetland. FEMS Microbiol Ecol 67: 308-319.

5. Baker GC, Smith JJ, Cowan DA (2003) Review and re-analysis of domain-specific 16S primers. J Microbiol Methods 55: 541-555.
